# A multiple comparison procedure for dose‐finding trials with subpopulations

**DOI:** 10.1002/bimj.201800111

**Published:** 2019-09-23

**Authors:** Marius Thomas, Björn Bornkamp, Martin Posch, Franz König

**Affiliations:** ^1^ Novartis Pharma AG Novartis Campus Basel Switzerland; ^2^ Section of Medical Statistics Medical University of Vienna Vienna Austria

**Keywords:** MCP‐Mod, multiple testing, subgroup analyses, targeted therapies

## Abstract

Identifying subgroups of patients with an enhanced response to a new treatment has become an area of increased interest in the last few years. When there is knowledge about possible subpopulations with an enhanced treatment effect before the start of a trial it might be beneficial to set up a testing strategy, which tests for a significant treatment effect not only in the full population, but also in these prespecified subpopulations. In this paper, we present a parametric multiple testing approach for tests in multiple populations for dose‐finding trials. Our approach is based on the MCP‐Mod methodology, which uses multiple comparison procedures (MCPs) to test for a dose–response signal, while considering multiple possible candidate dose–response shapes. Our proposed methods allow for heteroscedastic error variances between populations and control the family‐wise error rate over tests in multiple populations and for multiple candidate models. We show in simulations that the proposed multipopulation testing approaches can increase the power to detect a significant dose–response signal over the standard single‐population MCP‐Mod, when the specified subpopulation has an enhanced treatment effect.

## 1 INTRODUCTION

Identifying subgroups of patients with an enhanced response to a new treatment has become an area of increased interest in the last few years. Hence, subgroup analyses are commonly performed across the complete clinical development process. In many cases, these analyses are performed in an exploratory fashion and a large number of possible subgroups are considered with the aim to find a patient population in which the treatment is particularly effective. For these scenarios, many novel subgroup identification and treatment effect estimation methods have been proposed (see, e.g., Foster, Taylor, & Ruberg, 2011; Lipkovich, Dmitrienko, Denne, & Enas, 2011; Liu, Sivaganesan, Laud, & Müller, 2017; Rosenkranz, 2016; Seibold, Zeileis, & Hothorn, 2016; Shen et al., 2016; Varadhan & Wang, 2016). An extensive overview over exploratory subgroup analysis methods is given in Lipkovich, Dmitrienko, and D'Agostini (2017).

When such an exploratory subgroup analysis results in a promising subgroup finding, this subgroup could then be used in a future trial. Alternatively, such a subgroup could be identified before the start of a trial based on clinical or pharmacological information. In these situation, it might be beneficial to plan the trial with the subgroup in mind and test for a treatment effect in both full population and subgroup. Depending on the result of the trial, there are then three possible positive outcomes: (a) the drug is shown to be efficacious for all patients, (b) the drug is shown to be efficacious only in the subgroup, or (c) the drug is shown to be efficacious for all patients but with an increased efficacy for patients in the subgroup. In the last scenario, there might be uncertainty about the effect in the full population because it could be driven by the large effect in the subgroup and the rest of the population could have negligible treatment effects. One can consider an additional test in the complement to avoid this issue.

For the design of trials, in which subpopulations are of interest, additional considerations have to be made regarding the power of the tests and possible trial designs like enrichment or adaptive designs. Some of these considerations regarding the power when testing in the whole population and a subgroup are discussed in Koch (1997) and Alosh and Huque (2009). Ondra et al. (2016) discussed several different enrichment and adaptive designs.

When tests are performed in multiple populations, multiplicity has to be taken into account to avoid an inflated type I error. It is possible to use correlation‐free approaches to control multiplicity, for example, Bonferroni or Holm corrections, or more complex stepwise procedures as discussed in Bretz, Maurer, Brannath, and Posch (2009). However, correlations can be explicitly calculated when tests are performed in the overall population and a subgroup, where the latter is a subset of the former. Using a parametric approach can be more efficient and show an increase in power over the aforementioned methods. Such parametric approaches are, for example, discussed in Alosh and Huque (2009) and Bretz et al. (2011). Rosenblum, Qian, Du, Qiu, and Fisher (2016) also discussed multiple testing strategies for enrichment designs with subpopulations.

Dose‐finding trials are routinely performed in Phase II of clinical development and in some cases Phase III trials also investigate several different doses of the same treatment. In dose‐finding trials, additional multiplicity arises through tests for multiple doses. A well‐established test taking into account multiplicity, when comparing multiple doses against a common control in such settings was proposed in Dunnett (1955) and weighted parametric test procedures, which take the dose–response relationship into account, were proposed in Bretz et al. (2011) and Xi, Glimm, Maurer, and Bretz (2017).

Subgroup identification methods in the context of dose–response trials have recently been introduced in Thomas, Bornkamp, and Seibold (2018) and a testing approach, which can be used for dose–response trials with a prespecified subgroup would be useful in practice as well. Weighted parametric testing procedures can be used to test in multiple populations in such settings, but require assumptions about the underlying dose–response models. Frequently, there might however be uncertainty about the underlying dose–response shape and thus it is unclear which dose–response model would be appropriate. In this paper, we want to propose an approach allowing for tests in multiple populations under uncertainty about the dose–response shape, which is based on the MCP‐Mod methodology (Bretz, Pinheiro, & Branson, 2005).

The MCP‐Mod approach combines multiple comparison procedures (MCPs) and modeling to analyze dose–response trials. MCP‐Mod consists of two parts. In the MCP step a test for a dose–response signal is performed for several prespecified candidate dose–response shapes. The contrasts for the tests are chosen to achieve optimal power for the tests. MCP‐Mod uses the joint distribution of the test statistics to control the family‐wise error rate (FWER) at a specified level, while accounting for the correlations between test statistics. In case of a normally distributed outcome, the joint distribution of the tests statistics is known and the correlation structure between the test statistics depends on the candidate shapes used. The more general case, which is described in Pinheiro, Bornkamp, Glimm, and Bretz (2014), extends the framework to nonnormal outcomes using the asymptotic distribution of the test statistics. If a dose–response signal is established under any of the considered shapes, one can continue to the second step, the modeling step. In the modeling (Mod) step candidate models, for which a significant dose–response signal was established in the MCP step, can then be fitted to the data and be used for dose estimation. Further details concerning the design and analysis of clinical trials with MCP‐Mod are discussed in Pinheiro, Bornkamp, and Bretz (2006) and Xun and Bretz (2017).

The approach we propose in this paper is an extension of the MCP‐part of the MCP‐Mod methodology to allow for testing for a dose–response signal in multiple populations. We specifically focus on the situation of a Phase II trial, where there is uncertainty about the underlying dose–response shape and a subgroup with a possibly enhanced treatment effect has been prespecified. The proposed methods adjust for the multiplicity resulting from tests for different dose–response shapes and tests in multiple populations, and use the correlation between the tests. We also take into account that variance might differ between populations. Although we focus on tests in one subgroup and the full population, in general the methodology presented here can be used to perform multiple comparisons across arbitrarily overlapping populations of any number, while controlling the FWER.

The remainder of this paper is structured as follows. In the following section, we present a motivating example. In Section 3, we present the methodology to perform contrast tests in multiple populations. Section 4 shows the results of simulations, which evaluate the operating characteristics of the approach, focusing on FWER control and power. The paper concludes with a summary and discussion of results.

## 2 MOTIVATING EXAMPLE: MAVOGLURANT IN FRAGILE X SYNDROME

As a motivating example for the use of the presented methods, we consider clinical trials of mavoglurant in fragile X syndrome (FXS). FXS is a genetic condition and is generally considered to be the most common inherited cause of intellectual disability. FXS is caused by a mutation in the FMR1‐gene on the X‐chromosome. The mutation leads to complete or partial methylation (PM) of the FMR1 promoter, which causes a complete loss or a reduction in the expression of the associated FMRP (fragile X mental retardation protein; Berry‐Kravis et al., 2016).

Mavoglurant is a mGluR‐receptor antagonist and was considered to be a possible treatment option for FXS. In a small early‐phase efficacy clinical trial, there were overall no significant differences in efficacy between placebo and mavoglurant, however there was an improvement for a subgroup with patients with complete methylation (CM) of the FMR1 promoter. Based on these results phase 2b studies were conducted, which evaluated several doses of mavoglurant in patients with CM or PM (Berry‐Kravis et al., 2016).

In one of these phase 2b studies, three doses of mavoglurant were compared to placebo in adolescent FXS patients. One hundred thirty‐nine patients were stratified by methylation status and randomized to placebo, 25, 50, or 100 mg of mavoglurant. The primary efficacy endpoint was the change from baseline in Aberrant Behavior Checklist—Community Edition (ABC‐C) total score. The ABC‐C score consists of 58 items, each with a score of 0–3, thus total score ranges between 0 and 174. As it was suspected, that the treatment is more efficacious in the subgroup of CM patients, the primary objective of the study was to assess efficacy in this subgroup, however a key secondary endpoint was the efficacy in the complement of this subgroup, the PM group. Tests for a significant treatment effect were conducted for each of the three active doses in the CM and PM groups, so that in total six null hypotheses were tested. A graphical test procedure was used to control FWER over all tests.

As an alternative to the graphical test procedure, which tested each dose separately, multiple contrast tests could be used to test for a significant dose–response signal over all doses, while taking model uncertainty about the possible dose–response into account. Additionally, in this particular example it would also be of interest to test for such a dose–response signal in several populations. The approach we present in the following allows using a single‐step test to detect a significant dose–response signal in one of the populations of interest, either in the full population of CM and PM patients or only in the subgroup of CM patients. Compared to the originally used graphical test procedure, this allows for the possibility of rejecting in the full population, even if there is no significant dose–response signal in the subgroup.

## 3 MULTIPLE CONTRAST TESTS FOR MULTIPLE POPULATIONS

### 3.1 Notation, models, and contrast tests

We consider a clinical trial, which has been performed in parallel groups of patients, receiving different doses $d_{1},d_{2},\ldots ,d_{k}$ of an experimental treatment with $d_{1}=0$, that is, a placebo. The number of patients in the dose groups is $n_{1},\ldots ,n_{k}$ and $n:=\sum_{i=1}^{k}n_{i}$, the total number of patients in the study. We observe responses ***Y***, which can either be related to efficacy or safety of the new treatment. In the following, we distinguish between three populations of interest: the full or overall population (*F*), which includes all patients, and two prespecified subpopulations of *F*; a subpopulation of patients, which has been identified to possibly show an enhanced treatment effect (*S*) and the complement of that subpopulation (*C*), so that F=S∪C. The subgroup could be based on biomarkers or demographic or clinical covariates, such as a disease subtype. In what follows, we assume that the patients can be classified into subgroup and complement without error.

To simplify the discussion of the methodology we introduce two index sets, *U*, which contains the populations, in which contrast tests are performed and V:={S,C}, which is just used to define the underlying dose–response models in the two subpopulations. In this paper, we consider $U_{1}=\left\{F\right\}$, $U_{2}=\left\{F,S\right\},$ and $U_{3}=\left\{F,S,C\right\}$. Using *U*_1_ results in a single‐population (SP) testing strategy, as it is possible with the standard MCP‐Mod. In comparison *U*_2_ allows detecting a significant dose–response signal in the subgroup, if the overall effect is not significant. Finally, *U*_3_ results in a testing strategy, where tests are performed in all populations. The benefit of using *U*_3_ over *U*_2_ is the additional possibility to detect a significant signal in the complement. With *U*_2_ there can be uncertainty about the effect in the complement, even when a significant effect is detected in the full population, as this might be driven by a strong effect in *S*. In the rest of this section, we focus on $U=U_{3}$ as it is the most complete case and other testing strategies can be easily derived from it.

We adopt the general framework from Bretz et al. (2005), but additionally introduce the population as an additional component in the models. Analogous to the original MCP‐Mod framework, we therefore consider the model

(1) $$\begin{array}{ccc} Y_{ij}^{\left(P\right)} & = & f{\left(d_{i},\theta \right)}^{\left(P\right)}+\varepsilon_{ij}^{\left(P\right)};\varepsilon_{ij}^{\left(P\right)}∼N\left(0,\sigma_{P}^{2}\right)\;\text{i.i.d.},\\ & & i=1,\ldots ,k,j=1,\ldots ,n_{i}^{\left(P\right)},P\in V, \end{array}$$

where ***θ*** refers to the vector of model parameters, *i* to the dose group, and *j* refers to the patient within dose group *i* and population *P*. $n_{i}^{\left(P\right)}$ denotes the number of patients in population *P*, which are in dose group *i*, so that $n_{i}=n_{i}^{\left(F\right)}=n_{i}^{\left(S\right)}+n_{i}^{\left(C\right)}$. We assume homoscedasticity over the dose groups but allow for heteroscedasticity between populations with population‐specific variances $\sigma_{P}^{2}$. To simplify the discussion, we assume that the prevalence of the subgroup is deterministic and constant across all dose levels. We denote the prevalence of *S* by $\gamma :=\frac{n_{1}^{\left(S\right)}}{n_{1}^{\left(F\right)}}=\cdots =\frac{n_{k}^{\left(S\right)}}{n_{k}^{\left(F\right)}}$. We discuss more general scenarios (with more populations and non‐constant prevalences) in Section A1.

Model (1) is assumed to be the underlying dose–response model with regard to dose estimation. For the contrast tests, which have the aim of detecting a dose–response relationship, a different model,

(2) $$\begin{array}{ccc} Y_{ij}^{\left(P\right)} & = & \mu_{i}^{\left(P\right)}+\varepsilon_{ij}^{\left(P\right)};\varepsilon_{ij}^{\left(P\right)}∼N\left(0,\sigma_{P}^{2}\right)\;\text{i.i.d.},\\ & & i=1,\ldots ,k,j=1,\ldots ,n_{i}^{\left(P\right)},P\in V \end{array}$$

is considered. $\mu_{i}^{\left(P\right)}$ is the mean response for dose group *i* in population *P*. An estimate for $\mu_{i}^{\left(P\right)}$ is the arithmetic mean ${\bar{Y_{i}}}^{\left(P\right)}=\frac{1}{n_{i}^{\left(P\right)}}\sum_{j=1}^{n_{i}^{\left(P\right)}}Y_{ij}^{\left(P\right)}$. For population *P*, we denote the vector of the estimated dose means by ${\bar{Y}}^{\left(P\right)}={\left({\bar{Y_{1}}}^{\left(P\right)},\cdots ,{\bar{Y_{k}}}^{\left(P\right)}\right)}^{{}^{\prime }}$. Additionally, the pooled variance estimator for $\sigma_{P}^{2}$ is

(3) $${{\hat{\sigma }}_{P}}^{2}=\sum_{i=1}^{k}\sum_{j=1}^{n_{i}^{\left(P\right)}}{\left(Y_{ij}^{\left(P\right)}-{\bar{Y_{i}}}^{\left(P\right)}\right)}^{2}/\sum_{i=1}^{k}\left(n_{i}^{\left(P\right)}-k\right).$$

We assume uncertainty regarding the underlying shape of the dose–response relationship, and assume that a set of plausible candidate models $\left\{M_{1},\cdots ,M_{r}\right\}$ is prespecified. These models are candidates to describe the shape of the dose–response curve and produce mean vectors under the alternative. Usually, candidate models are commonly encountered dose–response models such as $E_{max}$, linear, exponential, or quadratic shapes. These candidate models are used to obtain the contrast vectors for the tests. Contrast vectors ${c_{m}}^{{}^{\prime }}=\left(c_{m1},\cdots ,c_{mk}\right)$ are chosen optimally to guarantee maximal power for each single contrast test. For details on choosing the optimal contrasts, see Bretz et al. (2005).

The MCP‐Mod approach then tries to detect a significant dose–response relationship for at least one of the models. In theory, it is possible to specify a different set of candidate shapes for each population. We assume here that the set of candidate models is the same across all populations, which is a plausible approach for most real‐life situations. With this assumption and the assumption of a constant prevalence of the subgroups across all dose groups, the optimal contrast vectors are independent of the population, in which the test is performed. We discuss the more general situation of different candidate models across populations in the Appendix.

We propose a single‐step procedure, in which for each candidate model $m\in \{M_{1},\cdots ,M_{r}\}$ and each population P∈U, a contrast test is performed to test the null hypothesis $H_{0}^{\left(P,m\right)}:{c_{m}}^{{}^{\prime }}\mu^{\left(P\right)}=0$ against the alternative $H_{1}^{\left(P,m\right)}:{c_{m}}^{{}^{\prime }}\mu^{\left(P\right)}>0$. Using this alternative we assume, without loss of generality, that the treatment effect will have a positive sign. After rejection of these individual null hypotheses $H_{0}^{\left(P,m\right)}$, we could conclude that a significant dose–response signal has been detected in population *P* for candidate model *m*.

We can further define the *population null hypothesis* (*in* *P* *)* as

$$H_{0}^{\left(P\right)}:H_{0}^{\left(P,M_{1}\right)}\cap \cdots \cap H_{0}^{\left(P,M_{r}\right)},$$

the intersection of the null hypotheses for all candidate models in population *P*. If this population null is rejected, we can conclude that a significant dose–response signal has been detected for at least one candidate model. The *global null hypothesis* is then the intersection between the population null hypotheses in all tested populations. For example, for the full testing strategy, which includes both subgroup and complement, the global null hypothesis is

$$H_{0}^{\left(global\right)}:H_{0}^{\left(F\right)}\cap H_{0}^{\left(S\right)}\cap H_{0}^{\left(C\right)}.$$

As rejection of the global null requires rejection of at least one candidate model in at least one population, we can conclude that a significant dose–response signal has been established in at least one population, if the global null hypothesis is rejected. Although we assume that rejecting the global null is of main interest, the population hypotheses have to be considered to determine if a dose–response trend has been established in all populations or, for example, only in the subgroup.

The test statistics for individual contrast tests are of the form

(4) $$T_{m}^{\left(P\right)}=\frac{{\left(c_{m}\right)}^{{}^{\prime }}{\bar{Y}}^{\left(P\right)}}{\sqrt{{{\hat{\sigma }}_{P}}^{2}\sum_{i=1}^{k}c_{mi}^{2}/n_{i}^{\left(P\right)}}}\;m\in \left\{M_{1},\cdots ,M_{r}\right\},P\in U.$$

If the variances in subgroup and complement are assumed to be the same, that is, we assume homoscedasticity between populations ($\sigma_{S}^{2}=\sigma_{C}^{2}=\sigma^{2}$), we can drop the population indices for the variances. Then it is also appropriate to drop the population index from the variance estimator in (4) and use the same estimator for all tests. A pooled variances estimator (pooled over dose levels and over subgroup and complement) is the natural choice in this context because model (2) assumes that the means are different both across dose levels, and subgroup and complement. Thus, we would replace ${{\hat{\sigma }}_{P}}^{2}$ in (4) with

(5) $${\hat{\sigma }}_{pooled}^{2}=\sum_{P\in V}\sum_{i=1}^{k}\sum_{j=1}^{n_{i}^{\left(P\right)}}{\left(Y_{ij}^{\left(P\right)}-{\bar{Y_{i}}}^{\left(P\right)}\right)}^{2}/\sum_{P\in V}\left(\sum_{i=1}^{k}n_{i}^{\left(P\right)}-k\right).$$

The assumption of homogeneous variances in all populations may not always be justified. For example, patients in the subgroup might be assumed to be more homogeneous than patients in the full population and thus the subgroup will be assumed to have smaller variances. If we assume the variances in subgroup and complement to be unequal, that is, we assume heteroscedasticity between subgroup and complement ($\sigma_{S}^{2}\neq \sigma_{C}^{2}$), the pooled variance estimator will generally be biased and it is more appropriate to estimate the variance separately in subgroup and complement. For the tests in subgroup and complement a variance estimate, which is pooled over the dose levels as in (3) can then be used.

Under the global null hypothesis, there is no difference between contrasts in subgroup and complement (both are assumed to be zero). Under these assumptions, a weighted average of the two population variance estimates is a reasonable estimator for the variance in the full population. For the tests in the full population under assumed heteroscedasticity, we would therefore use the variance estimator ${{\hat{\sigma }}_{F}}^{2}=\frac{1}{n}\cdot \left[\sum_{i=1}^{k}n_{i}^{\left(S\right)}{{\hat{\sigma }}_{S}}^{2}+\sum_{i=1}^{k}n_{i}^{\left(C\right)}{{\hat{\sigma }}_{C}}^{2}\right]$.

### 3.2 FWER control

Performing multiple tests at once, as in the approach we described above, leads to multiplicity issues. In the classic MCP‐Mod approach, the FWER across all tests is controlled, for example, the probability to reject at least one true null hypothesis is below a specified level α. The MCP‐Mod approach uses the joint distribution of the test statistics to obtain critical values or adjusted *p*‐values. In the situation we consider here, in addition to the multiplicity stemming from testing different candidate shapes, there is also multiplicity stemming from tests in several populations. When MCP‐Mod is performed in only one population, the joint distribution is known to be multivariate *t* under the assumptions of model (2). When considering multiple populations, the joint distribution of the test statistics is only multivariate *t*, if the variance is assumed to be equal across all populations.

In the following discussion, we assume that tests are performed in the full population, subgroup, and complement because this is the most complete testing strategy and the distributions for other strategies can easily be derived from this.

We will first consider the homoscedastic case, where variances are assumed to be equal in subgroup and complement ($\sigma_{S}^{2}=\sigma_{C}^{2}=\sigma^{2}$). Then it is appropriate to use the pooled variance estimate (3) in all populations and the vector of test statistics $T^{{}^{\prime }}=\left(T_{M_{1}}^{\left(F\right)},\cdots ,T_{M_{r}}^{\left(F\right)},T_{M_{1}}^{\left(S\right)},\cdots ,T_{M_{r}}^{\left(S\right)},T_{M_{1}}^{\left(C\right)},\cdots ,T_{M_{r}}^{\left(C\right)}\right)$ is distributed as $MVT_{3r}\left(\nu ,0,R\right)$ with $\nu =\sum_{P\in V}\left(\sum_{i=1}^{k}n_{i}^{\left(P\right)}-k\right)$ degrees of freedom, mean vector 0 and correlation matrix ***R***. The correlation matrix has the form

$$R=\left[\begin{array}{ccc} R_{\mathrm{FF}} & R_{\mathrm{FS}} & R_{\mathrm{FC}}\\ R_{\mathrm{FS}}^{{}^{\prime }} & R_{\mathrm{SS}} & R_{\mathrm{SC}}\\ R_{\mathrm{FC}}^{{}^{\prime }} & R_{\mathrm{SC}}^{{}^{\prime }} & R_{\mathrm{CC}} \end{array}\right],$$

where the entries of submatrix $R_{\mathrm{PQ}}\in R^{r\times r}$ for P,Q∈U are given as

$${\left(\rho_{ij}\right)}^{\left(PQ\right)}=\frac{\sum_{l=1}^{k}c_{il}c_{jl}\cdot n_{l}^{\left(P\cap Q\right)}/\left(n_{l}^{\left(P\right)}n_{l}^{\left(Q\right)}\right)}{\sqrt{\sum_{l=1}^{k}{c_{il}}^{2}/n_{l}^{\left(P\right)}\sum_{l=1}^{k}{c_{jl}}^{2}/n_{l}^{\left(Q\right)}}},\;i,j=1,\cdots ,r.$$

Based on the above formula, it is clear that ***R_SC_*** = ***0*** because subgroup and complement are distinct. The other submatrices are easily obtainable as well, for example,

$${\left(\rho_{ij}\right)}^{\left(FS\right)}=\sqrt{\gamma }\cdot \frac{\sum_{l=1}^{k}c_{il}c_{jl}/n_{l}^{\left(F\right)}}{\sqrt{\sum_{l=1}^{k}{c_{il}}^{2}/n_{l}^{\left(F\right)}\sum_{l=1}^{k}{c_{jl}}^{2}/n_{l}^{\left(F\right)}}},\;i,j=1,\cdots ,r$$

follows for the entries of the matrix ***R_FS_***.

The joint distribution for performing tests only in the full population and in the subgroup without a separate test in the complement can easily be derived by dropping the irrelevant test statistics from the vector $T^{{}^{\prime }}$ and removing the irrelevant submatrices (in this case the last row and column) from ***R***.

The joint distribution can then be used to obtain a multiplicity‐adjusted critical value $q_{1-\alpha }$, such that $P_{H_{0}^{\left(global\right)}}\left(T_{M_{1}}^{\left(F\right)}\leq q_{1-\alpha },\cdots ,T_{M_{r}}^{\left(F\right)}\leq q_{1-\alpha },T_{M_{1}}^{\left(S\right)}\leq q_{1-\alpha },\cdots ,T_{M_{r}}^{\left(S\right)}\leq q_{1-\alpha },T_{M_{1}}^{\left(C\right)}\leq q_{1-\alpha },\cdots ,T_{M_{r}}^{\left(C\right)}\leq q_{1-\alpha }\right)=1-\alpha$ to guarantee strong control of the FWER over the population null hypotheses. Then all hypotheses $H_{0}^{\left(P,m\right)}$, for which the corresponding test statistics are larger than $q_{1-\alpha }$, can be rejected.

If variances cannot be assumed to be equal in subgroup and complement the variance estimator in the denominator of the test statistics, (4) is population‐dependent, and the degrees of freedoms of the *t*‐distributions are no longer the same in all populations. Therefore, the joint distribution is not multivariate *t* and in fact does not seem to belong to any standard probability distribution. The terms in the correlation matrix ***R*** also become more complex because the variance terms no longer cancel out for all of the submatrices. In general, the correlation submatrices then have entries

$$\begin{array}{ccc} & & {\left(\rho_{ij}\right)}^{\left(PQ\right)}=\frac{\sum_{l=1}^{k}c_{il}c_{jl}\cdot \left(n_{l}^{\left(P\cap Q\cap S\right)}\sigma_{S}^{2}+n_{l}^{\left(P\cap Q\cap C\right)}\sigma_{C}^{2}\right)/\left(n_{l}^{\left(P\right)}n_{l}^{\left(Q\right)}\right)}{\sqrt{\sum_{l=1}^{k}\left\{{c_{il}}^{2}\cdot \left(n_{l}^{\left(P\cap S\right)}\sigma_{S}^{2}+n_{l}^{\left(P\cap C\right)}\sigma_{C}^{2}\right)/{\left(n_{l}^{\left(P\right)}\right)}^{2}\right\}\sum_{l=1}^{k}\{{c_{jl}}^{2}\cdot \left(n_{l}^{\left(Q\cap S\right)}\sigma_{S}^{2}+n_{l}^{\left(Q\cap C\right)}\sigma_{C}^{2}\right)/{\left(n_{l}^{\left(Q\right)}\right)}^{2}}\}},\\ & & i,j=1,\cdots ,r;P,Q\in U. \end{array}$$

For example, for the entries of submatrix ***R_FS_*** we get

$${\left(\rho_{ij}\right)}^{\left(FS\right)}=\frac{\sigma_{S}\sum_{l=1}^{k}c_{il}c_{jl}/n_{l}^{\left(F\right)}}{\sqrt{\left[\gamma \sigma_{S}^{2}+\left(1-\gamma \right)\sigma_{C}^{2}\right]\sum_{l=1}^{k}{c_{il}}^{2}/n_{l}^{\left(F\right)}\sum_{l=1}^{k}{c_{jl}}^{2}/n_{l}^{\left(S\right)}}},\;i,j=1,\cdots ,r.$$

The variances $\sigma_{S}^{2}$ and $\sigma_{C}^{2}$ are usually unknown. To obtain the correlation matrix, the estimates for $\sigma_{S}^{2}$ and $\sigma_{C}^{2}$ can be plugged into the formulas above.

There are several reasonable options to approximate the joint distribution of the test statistics under heteroscedasticity and obtain multiplicity‐adjusted *p*‐values. A simple solution is to assume that the variances are known and use the multivariate normal distribution to obtain *p*‐values and critical values. As the multivariate *t* distribution approaches a multivariate normal distribution as n→∞, this approximation will work well for reasonably large sample sizes.

For small sample sizes, a conservative option is to use the smallest degrees of freedom across all populations, $\nu_{min}:=\min_{P\in U}\nu^{\left(P\right)}$, where $\nu^{\left(P\right)}:=\sum_{i=1}^{k}n_{i}^{\left(P\right)}-k$ are the population‐specific degrees of freedom. In the considered setting, this is equivalent to using the degrees of freedom from the population with the smallest number of patients. Then an $MVT_{3r}\left(\nu_{min},0,R\right)$ distribution can be used as an approximate joint distribution.

A less conservative approximation, which is proposed in Hasler (2014) is to use a *multiple degrees of freedom* approximation, which translates to using a different multivariate *t*‐distribution with the correct degrees of freedom for each population to obtain critical values. For example, for test statistics from population P∈U, $T_{M_{1}}^{\left(P\right)},\cdots ,T_{M_{r}}^{\left(P\right)}$, the critical values are obtained from a $MVT_{3r}\left(\nu^{\left(P\right)},0,R\right)$ distribution. A similar approach has also been discussed by Graf, Wassmer, Friede, Gera, and Posch (2019) for standard (noncontrast) *t*‐tests in subgroups, while additionally allowing for nonfixed sample sizes in subgroups.

Table 1 summarizes all discussed methods both under the assumption of homoscedasticity and heteroscedasticity.

**Table 1:** Overview over considered MCPs with the variance estimates used in the test statistics, the distribution used to obtain critical values and the degrees of freedom for the multivariate *t* distributions. Here $\nu^{\left(P\right)}:=\sum_{i=1}^{k}n_{i}^{\left(P\right)}-k$, the degrees of freedom for population *P*

Method	Description	Variance estimation	Distribution	Degrees of freedom
SP	Standard MCP in single (full) population	Pooled	Multivariate *t*	$\sum_{i=1}^{k}n_{i}^{\left(F\right)}-k$
MP‐Pooled	Multipopulation MCP assuming homoscedasticity	Pooled	Multivariate *t*	$\sum_{P\in V}\sum_{i=1}^{k}\nu^{\left(P\right)}$
MP‐MinDF	Multipopulation MCP assuming heteroscedasticity	Population specific	Multivariate *t* (approx.)	$\min_{P\in U}\nu^{\left(P\right)}$
MP‐MultDF	Multipopulation MCP assuming heteroscedasticity	Population specific	Multivariate *t* (approx.)	$\nu^{\left(P\right)}$ (different for each population)
MP‐Normal	Multipopulation MCP assuming heteroscedasticity	Population specific	Multivariate normal (approx.)	–

Abbreviations: MCP, multiple comparisons procedure; MP, multipopulation; SP, single population.

### 3.3 Dose estimation

A key aspect of Phase II dose‐finding trials is dose estimation. In the Mod step of MCP‐Mod, a target dose, for example, the minimally effective dose can be estimated based on the significant models from the MCP‐step. For this purpose, model averaging can be used to take model uncertainty into account, if contrast tests were significant for several candidate models.

In the setting with multiple populations, we can use a similar approach to estimate doses in one or multiple populations. If only one of the population null hypotheses can be rejected, we propose to simply fit all significant candidate models in that population only and use model averaging to estimate a dose.

If more than one population null can be rejected, the situation becomes more complex because the different populations should be taken into account for dose estimation. We propose to use a model averaging approach that is motivated by Bornkamp et al. (2017) who use model averaging for treatment effect estimation for subgroups. Similar to their approach, we use interaction effects for subgroups to model different dose–response curves in populations.

For the model averaging all candidate shapes, for which $H_{0}^{\left(P,m\right)}$ could be rejected for any P∈U, could be considered in the Mod‐step. Thus, as long as there is a significant dose–response signal for a candidate shape in any of the populations, it is used in the Mod step. We denote the number of candidate shapes for which a significant signal could be declared in the MCP step by $r^{*}$.

We then propose to use model averaging over two sets of dose–response models. In the first set, dose–response models are fit in the full population only and differences between populations are not taken into account. The second set includes subgroup‐treatment interactions to account for differential dose–response in subgroup and complement.

In detail, we propose to average over the $2r^{*}$ models

(6) $$\begin{array}{ccc} M_{m^{*}}:Y_{ij} & = & f_{m^{*}}\left(d_{i},\theta \right)+\varepsilon_{ij};\varepsilon_{ij}∼N\left(0,\sigma^{2}\right)\;i.i.d,\\ & & i=1,\ldots ,k,j=1,\ldots ,n_{i},m^{*}=1,\ldots ,2r^{*} \end{array}$$

with

$$f_{m^{*}}\left(d,\theta \right)=\left\{\begin{array}{c} \theta_{0}+\theta_{1}f_{m^{*}}^{0}\left(d,\theta \right),\;\text{for}\;m^{*}=1,\cdots ,r^{*}\\ \theta_{0}+\left(\theta_{1}+\delta s\right)f_{m^{*}}^{0}\left(d,\theta \right),\;\text{for}\;m^{*}=r^{*}+1,\cdots ,2r^{*}. \end{array}\right.$$

Here *f* ^0^ denote standardized forms of the candidate models (see also Table 2) and *s* is a binary variable, indicating whether a patient is in the subgroup (s=1) or in the complement. Thus, the second set of models assumes different “slopes” (e.g., different $E_{max}$) in subgroup and complement. The general form for the model with subgroup‐treatment interaction holds for most common dose–response models, however for a quadratic model there is not just a single slope parameter. For a quadratic model, we would therefore use $f_{m}\left(d,\theta \right)=\theta_{0}+\left(\theta_{1}+\delta_{1}s\right)d+\left(\theta_{2}+\delta_{2}s\right)d^{2}$.

**Table 2:** Data‐generating dose–response models and corresponding standardized versions, which are used as candidate shapes to determine optimal contrast tests

Model	f(d,θ)	Standardized model	Fixed parameters
Constant	0.2	–	–
$E_{max}$	$\theta_{0}+\frac{\theta_{1}d}{\left(\theta_{2}+d\right)}$	$\frac{d}{\left(\theta_{2}+d\right)}$	$\theta_{0}=0.2$; $\theta_{2}=0.2$
Linear	$\theta_{0}+\theta_{1}d$	*d*	$\theta_{0}=0.2$
Exponential	$\theta_{0}+\theta_{1}\cdot exp\left[\frac{d}{\theta_{2}}-1\right]$	$exp[\frac{d}{\theta_{2}}-1]$	$\theta_{2}=0.29$
Logistic	$\theta_{0}+\frac{\theta_{1}}{1+exp[\left(\theta_{2}-d\right)/\theta_{3}}$	$\frac{1}{1+exp[\left(\theta_{2}-d\right)/\theta_{3}}$	$\theta_{2}=0.4$; $\theta_{3}=0.091$
Quadratic	$\theta_{0}+\theta_{1}d+\theta_{2}d^{2}$	$d+\frac{\theta_{2}}{|\theta_{1}|}d^{2}$	θ1θ212=1.171

*Note*. Parameters specified in the last column are fixed for all simulated trials and are used as guesstimates to derive optimal contrasts.

Using model averaging over the above models target doses can be estimated for each population of interest, for example, for each population for which the population null hypothesis could be rejected. For details on model averaging for dose estimation, see, for example, Xun and Bretz (2017).

## 4 SIMULATION STUDY

In this section, we discuss results of a simulation study to evaluate the properties of the multipopulation (MP) testing approaches. The simulation setup is similar to the one used in Bretz et al. (2005). The simulations are divided into two main parts. In the first part (Section 4.2), we assume homoscedasticity across populations and evaluate the power of the proposed MP testing approaches compared to the standard SP MCP, which only considers the full population. The second part (Section 4.3) deals with the scenario of heteroscedastic populations. In addition to the comparison of MP MCP to SP MCP, the main focus of this section is to evaluate the different considered procedures, which are based on approximations of the joint distribution, with regard to FWER control and power.

### 4.1 Simulation setup

We simulate clinical trials with parallel groups, which are balanced over the dose levels. In our simulated trial, patients are randomized to one of five different dose groups with dose levels 0, 0.05, 0.2, 0.6, 1. We assume that there is knowledge about a subgroup, which possibly shows an enhanced treatment effect and could have been identified from an earlier trial or based on the mode of action of the drug. We also make the simplifying assumption that for each dose group, the proportion of patients in the subgroup is equal. Together with the assumption of a balanced trial design, this means that $n_{1}^{\left(S\right)}=\cdots =n_{k}^{\left(S\right)}$.

We generate the observed data for the simulated trials from models of the form

(7) $$\begin{array}{ccc} Y_{ij}^{\left(P\right)} & = & f{\left(d_{i},\theta \right)}^{\left(P\right)}+\varepsilon_{ij}^{\left(P\right)};\varepsilon_{ij}^{\left(P\right)}∼N\left(0,\sigma_{P}^{2}\right)\;i.i.d,\\ & & i=1,\ldots ,k,j=1,\ldots ,n_{i}^{\left(P\right)},P\in V \end{array}$$

and use common dose–response shapes for *f*. An overview of the used dose–response shapes is given in Table 2. We also include a constant model to generate data under the global null hypothesis.The subgroup we simulate has prevalences γ of either 0.25, 0.5, or 0.75. We assume that the underlying shape is the same for subgroup and complement. However the “slope” parameters of the model, which are not fixed (θ_1_ in Table 2), can vary between populations. This allows us to simulate scenarios with different treatment effects between subgroup and complement.

We consider three scenarios for how the treatment effect is distributed between subgroup and complement. In the first scenario, there is no enhanced effect in the subgroup, for example, the treatment effect is identical in the subgroup and the complement (*same*‐scenario). In the second scenario, the treatment effect in the subgroup is double the size of the effect in the complement (*double*‐scenario). Finally, for the third and last scenario, there is only a treatment effect in the subgroup and the treatment effect in the complement is zero (*only*‐scenario). The three different scenarios represent situations in which there is in reality no subgroup and possible treatment effects are constant across the full population (*same*), or in which a treatment effect in the full population is driven mostly (*double*) or completely (*only*) by a large effect in the subgroup.

We choose θ_1_ for the nonconstant models so that the treatment effect at the highest dose is 0.6, which leads to a power of 80% (for a pairwise comparison) for a group size of 75. For the *same*‐scenario 0.6 is the treatment effect in the full population, for the *only*‐scenario and *double*‐scenario it is the treatment effect in the subgroup. This leads to scenarios, where the treatment effect is relatively large in the subgroup but possibly hard to detect (depending on γ) in the full population.

For the MCP, we then perform tests for all nonconstant shapes in Table 2 in all populations we consider, irrespective of which of the shapes was used for data generation. For the MP MCP approaches, we compare two different testing strategies. These testing strategies differ with regard to the populations in which tests are performed. Tests are performed either in the full population and the subgroup (F+S) or in full population, subgroup, and the complement (F+S+C).

Source code to reproduce the results is available as Supporting Information at the journal's web page.

### 4.2 Homoscedastic scenarios

We will first discuss the simulation results for equal variances in subgroup and in the complement. For these simulations, we fix the standard deviations at $\sigma_{S}=\sigma_{C}=1.478$. In the paper, only selected results are shown. Complete simulation results for all data‐generating models are included in the Supporting Information.

In the homoscedastic case, the exact joint distributions of the test statistics can be used to obtain critical values or adjusted *p*‐values for the individual tests as described in Section 3.2. As the exact distribution of the test statistics is known, FWER is controlled at the nominal 5% level for all testing methods, when data are generated under the global null hypothesis, from a constant model. Power under different scenarios can also be calculated from the joint distribution. Simulations are however necessary to determine power when population‐specific variance estimation is used, as the joint distribution of test statistics can then only be approximated.

**Figure 1:**
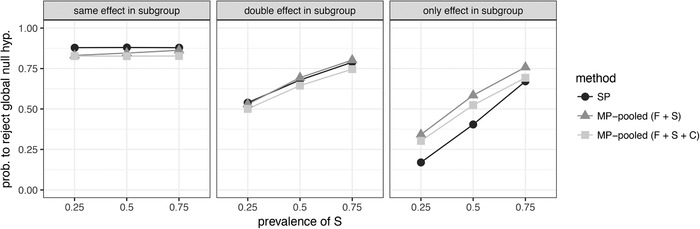
Probability to reject the global null hypothesis for single‐population (SP) and multipopulation (MP) testing methods *Note*. Data are generated from an $E_{max}$ model under homoscedasticity

Under the alternative of an existing dose–response trend, the MP testing approach with tests in full population and subgroup increases the chance of rejecting the global null hypothesis when the treatment effect only exists in the subgroup or is doubled in the subgroup. This is visualized for data generated from an $E_{max}$ model in Figure 1. The MP approach with tests in all populations (F + S + C) also shows more rejections than SP in the *only*‐scenario. In the *same*‐scenario, SP rejects most often, but the loss in power for the MP methods is relatively small, especially when the subgroup is large.

For the MP methods, it might additionally be of interest for which population(s) a significant dose–response relationship is declared. For example, a significant result only in the subgroup, but not in the full population or the complement, might lead to very different conclusions than significant results in all populations. Population‐specific rejection results for data generated from $E_{max}$ models are summarized in Table 3. The results show that when the treatment effect only exists in the subgroup, the null hypothesis in the subgroup is rejected up to 24% more often than the null hypothesis in the full population. The null hypothesis for the complement, which is true in the *only*‐scenario, is only rejected in 2% of trials. When the treatment effect in the subgroup is doubled over the complement, rejections generally occur more often in the full population than in the subgroup. The difference decreases with increasing subgroup prevalence γ. Table 3 also includes the results for the MP‐MultDF method, which would also allow for possible heteroscedasticity. The results are essentially the same as for MP‐Pooled, there does not seem to be a big penalty for allowing for heteroscedasticity in homoscedastic scenarios.

**Table 3:** Probability to reject the global null hypothesis and population null hypotheses for single‐population (SP) and multipopulation (MP) testing methods

		Scenario and prevalence								
		Same	Same	Same	Double	Double	Double	Only	Only	Only
Method	Hypothesis	0.25	0.5	0.75	0.25	0.5	0.75	0.25	0.5	0.75
SP	Global	0.88	0.88	0.88	0.54	0.68	0.79	0.17	0.41	0.67
MP‐Pooled(F + S)	Global	0.83	0.85	0.86	0.53	0.69	0.80	0.34	0.59	0.76
	F	0.82	0.82	0.84	0.43	0.59	0.74	0.11	0.32	0.60
	S	0.30	0.54	0.73	0.30	0.54	0.73	0.30	0.54	0.72
MP‐Pooled(F + S + C)	Global	0.83	0.83	0.83	0.50	0.64	0.75	0.30	0.52	0.69
	F	0.79	0.78	0.79	0.39	0.53	0.66	0.09	0.26	0.53
	S	0.26	0.48	0.66	0.26	0.48	0.65	0.26	0.48	0.66
	C	0.65	0.47	0.27	0.20	0.14	0.09	0.02	0.02	0.02
MP‐MultDF(F + S)	Global	0.83	0.85	0.86	0.53	0.70	0.80	0.34	0.58	0.76
	F	0.82	0.83	0.84	0.43	0.59	0.73	0.11	0.32	0.61
	S	0.29	0.54	0.73	0.30	0.54	0.72	0.29	0.54	0.73
MP‐MultDF(F + S + C)	Global	0.83	0.83	0.83	0.50	0.64	0.75	0.30	0.52	0.69
	F	0.79	0.78	0.79	0.39	0.53	0.67	0.10	0.26	0.52
	S	0.26	0.47	0.66	0.26	0.48	0.65	0.26	0.48	0.66
	C	0.65	0.47	0.26	0.20	0.15	0.09	0.02	0.02	0.02

*Note*. Data are generated from an $E_{max}$ model under homoscedasticity.

One of the main features of the discussed methods is the possibility to test for several possible dose–response shapes. Hence, it is also of interest to compare the model selection performance of the MP testing approaches compared to the SP‐MCP. Table A.1 shows the probability of choosing the correct model for the different methods. The probability of choosing the correct model based on the lowest significant *p*‐value is almost identical between SP and MP approaches, and therefore testing in multiple populations does not seem to reduce the model selection performance.

### 4.3 Heteroscedastic scenarios

In this part we will show simulation results, when testing in multiple populations with different variances. Here, we use $\sigma_{S}=1.03$ and $\sigma_{C}=1.926$ for the standard deviations in subgroup and complement. The combined standard deviation in the full population thus remains the same as before at 1.478, as long as subgroup and complement have equal prevalence (γ=0.5).

As discussed in Section 3.2, the joint distribution of the contrast test statistics does not follow a standard distribution when variances are heterogeneous, and instead approximations to the true joint distribution are necessary to obtain critical values. In these simulations, we will therefore first compare the different possible approximations we discuss in Section 3.2. We focus on FWER control and power for the considered approximations. For each of the scenarios, we simulate 25,000 trials.

The approaches we consider for approximating the joint distribution are MP‐MinDF, MP‐MultDF, and MP‐Normal (see Table 1). All these approaches use separate variance estimates for the tests in subgroup and complement. To evaluate the general necessity of using population‐specific variance estimates, we also include test statistics using the pooled variance estimate (MP‐Pooled), which assumes that variances are the same in subgroup and complement.

**Figure 2:**
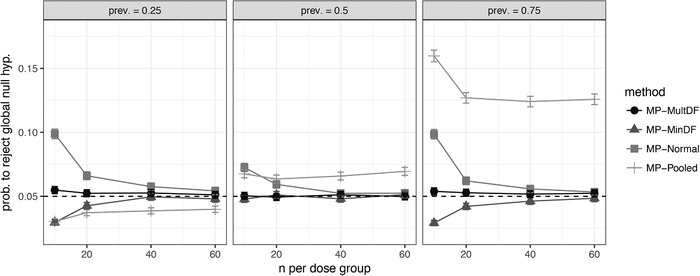
Probability to reject the global null hypothesis for different multipopulation (MP) testing methods *Note*. Data are generated from a constant model under heteroscedasticity. Tests were performed in full population, subgroup, and complement. Bars show the 95% confidence intervals (accounting for simulation error)

Figure 2 shows the probability of rejecting the global null hypothesis when the global null is true, for example, data are generated from a constant model. MP‐Pooled, which ignores heteroscedasticity, generally does not control the FWER at the nominal level because the variance in the complement is underestimated when the subgroup is large. For the subgroup with prevalence of 0.75, the FWER is generally above 0.1 with the pooled variance estimate. Of the three approximations, which use population‐specific variance estimates, MP‐Normal is as expected liberal and leads to a large inflation of the FWER for small sample sizes. For large sample sizes MP‐Normal approximates the true joint distribution better and the FWER is close to the nominal level. Of the two remaining approaches, which use approximate multivariate *t* distributions, the MP‐MinDF approach controls the FWER rate at the nominal level for all sample sizes and prevalences. MP‐MultDF shows similar error rates but shows FWERs slightly higher than 0.05 for very small sample sizes.

**Figure 3:**
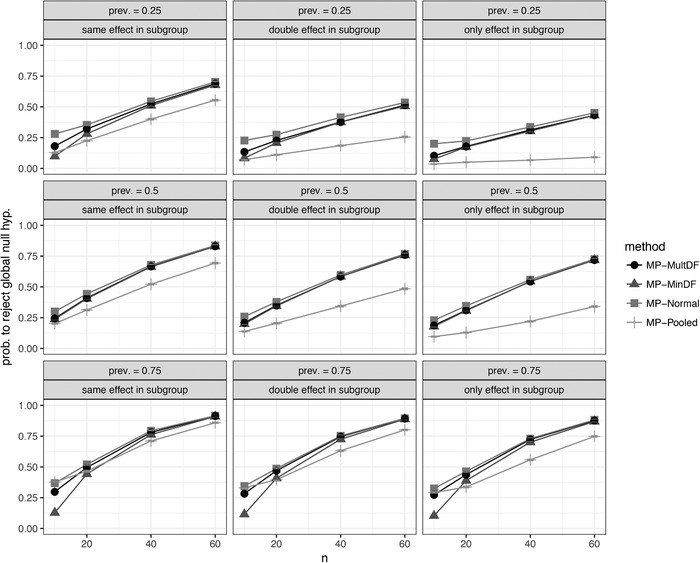
Probability to reject the global null hypothesis for different multipopulation (MP) testing methods *Note*. Data are generated from an $E_{max}$ model under heteroscedasticity. Tests were performed in full population, subgroup, and complement

Figure 3 visualizes the differences with regard to power, for example, the frequency of rejections of the global null hypothesis for data generated from an $E_{max}$ model for different scenarios and subgroup prevalences. With pooled variance estimation, the power is generally reduced compared to other methods. The approaches using population‐specific variances estimates show fairly similar power, with MP‐Normal being more liberal for small sample sizes and therefore rejecting more often. The MP‐MultDF approach always leads to slightly more rejections than the MP‐MinDF approach. The conclusions for the other data‐generating dose–response models are similar, detailed results for these models can be found in the Supporting Information.

**Figure 4:**
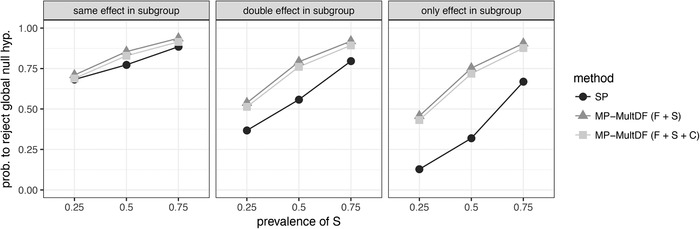
Probability to reject the global null hypothesis for single‐population (SP) and multipopulation (MP) testing methods *Note*. Data are generated from an $E_{max}$ model under heteroscedasticity. MP‐MultDF is used to approximate the joint distribution for MP testing methods

Finally, it is of interest to compare the MP testing strategies to the SP MCP in the heteroscedastic setting as well. We focus here on the MP‐MultDF approach because it gives FWER control roughly at the nominal level, while not sacrificing too much power. Figure 4 compares the methods for a sample size of 60 per group. Comparing Figure 4 to Figure 1, the differences are now even larger between SP and MP methods. As SP does not account for the different variances in subgroup and complement, the MP methods give better power even when there is no increased treatment effect in the subgroup and the treatment effect is the same for all patients.

## 5 DISCUSSION

In this paper, we discussed an extension of the MCP‐Mod methodology to allow for testing in multiple populations. We focused on the situation of a dose–response trial, where a subgroup with a suspected enhanced treatment effect has been prespecified. Our method allows for simultaneous testing in full population, subgroup, and possibly complement under model uncertainty, while controlling the FWER across all candidate models and populations.

Our simulation results in Sections 4.2 and 4.3 show that an increase in power of rejecting the global null hypothesis can be achieved when using the MP approach. The results suggest that the prevalence of the subgroup and the size of the treatment effect in the subgroup relative to the complement are important factors to determine if a MP approach is worth considering. Additional plots, which visualize the effects of these factors on the power in more detail, are included in the Supporting Information.

Instead of considering the power, one can also use sample size calculations to illustrate the potential benefits of a MP testing approach. Table 4 shows the sample sizes needed to achieve 80% power for rejecting the global null hypothesis under the scenarios considered for the simulation study in Section 4. For situations, where the subgroup has a strongly enhanced effect over the rest of the population, the sample sizes for the trial can be significantly reduced when using an MP approach instead of an SP approach.

**Table 4:** Sample sizes (per dose) required to achieve 80% power for rejecting the global null hypothesis for single‐population (SP) and multipopulation (MP) testing methods

	Scenario and prevalence								
	Same	Same	Same	Double	Double	Double	Only	Only	Only
Method	0.25	0.5	0.75	0.25	0.5	0.75	0.25	0.5	0.75
SP	59	60	59	151	105	77	932	235	105
MP‐Pooled(F + S)	70	67	63	151	101	75	275	131	84
MP‐Pooled(F + S + C)	71	70	70	163	112	87	299	147	97

*Note*. Required sample sizes are calculated under the assumption of homoscedasticity across populations.

In the heteroscedastic case, the simulation results discussed in Section 4.3 show that the multiple degrees of freedom approach seems to result in a good approximation to the joint distribution of the test statistics. The FWER inflation is minimal compared to the multivariate normal approximation, which is only suitable for large sample sizes. Additionally, the multiple degrees of freedom approach sacrifices less power than the conservative minimum degrees of freedom approach.

In the introduction, we stated three different outcomes for a trial in multiple populations: (a) the drug is shown to be efficacious for all patients, (b) the drug is shown to be efficacious only in the subgroup, or (c) the drug is shown to be efficacious for all patients but with an increased efficacy for patients in the subgroup. The methodology we present here can be used to help with decision making in this context. We would advise to use our methodology together with estimates of treatment effects in the different populations to decide which of the three outcomes is achieved. Millen, Dmitrienko, Ruberg, and Shen (2012) propose a decision framework for testing in a subgroup and the full population to arrive at one of the three conclusions.

We focused on scenarios, where a single subgroup and possibly the complement were considered for testing apart from the full population. Nevertheless, the methodology we present here is not restricted to this setting and can be used to test in an arbitrary number of populations. We extend the method to more general scenarios in Section A1. Although one could therefore include a large number of subgroups and use the presented methodology to identify subgroups with an enhanced effect, we would like to point out that in contrast to other subgroup identification approaches our method does not test for an interaction between subgroup and treatment. A significant result in the subgroup does therefore not necessarily indicate that the subgroup shows a differential effect and could be caused by a treatment effect that is large but constant across all populations. Still, there are certain outcomes, which can be indicative of a subgroup with a larger treatment effect, most notably, when the population null hypothesis for the subgroup is rejected but the population null hypothesis for the full population is not. In our simulations, this can be seen for some scenarios in Table 3. For scenarios, where there is only an effect in the subgroup, the null hypothesis for the subgroup is more often rejected than for the full population.

We only consider normally distributed outcomes in this paper. The MCP‐Mod methodology has been generalized to other types of outcomes in Pinheiro et al. (2014). It is possible to apply the presented methodology to general parametric models. In the general case of MCP‐Mod, the approximate multivariate normal distribution is used. We already use this approximation in this paper in Section 4.3 and show that it is a reasonable approximation for large sample sizes. The correlation matrices we discuss in Section 3.2 can simply be used for the multivariate normal instead of the multivariate *t* in the general case.

The testing procedure discussed here is a single‐step test. Alternatively, a sequentially rejective test using the closed testing principle could be used as well. A similar procedure is discussed for the single‐population MCP‐Mod in Bretz, König, and Bornkamp (2017). Furthermore, similarly as in Bretz et al. (2011), the multiple testing procedure can be generalized to a weighted test and extended to a graph‐based closed testing procedure.

## CONFLICT OF INTEREST

The authors declare that there is no conflict of interest.

## Supporting information

Supporting Information

Supporting Information

## APPENDIX 1

### A.1 Joint distribution in the general case

In this section, we derive the correlation matrix for the joint distribution of the contrast tests. We will derive the matrix for a general case with an arbitrary number of populations and also include the possibility that different candidate shapes are tested in each population. The scenarios described in Section 3.2 are special cases of what we discuss in this section.

We assume that we have *W* populations that we consider for testing. These populations are contained in the family $U:=\{P_{1},\cdots ,P_{W}\}$, where each population P∈U is a set of patients, so that P⊂{1,⋯,n}. As in the main part of the paper, we need to introduce a second family of pairwise disjoint populations $V:=\{P_{1}^{*},\cdots ,P_{W^{*}}^{*}\}$ so that ${⋃}_{r=1}^{W^{*}}P_{r}^{*}=\left\{1,\cdots ,n\right\}$, if we want to allow for populations with different variances. We then assume that our responses come from the model

(A.1) $$\begin{array}{ccc} Y_{ij}^{\left(P^{*}\right)} & = & \mu_{i}^{\left(P^{*}\right)}+\varepsilon_{ij}^{\left(P^{*}\right)};\varepsilon_{ij}^{\left(P^{*}\right)}∼N\left(0,\sigma_{P^{*}}^{2}\right)\;i.i.d,\\ & & i=1,\ldots ,k,j=1,\ldots ,n_{i}^{\left(P^{*}\right)},P^{*}\in V \end{array}$$

and the vector of responses is $Y=(Y_{11},\ldots ,Y_{1n_{1}},\ldots ,Y_{k1},\ldots ,Y_{kn_{k}})$ with $\text{cov}\left(Y\right):=\Sigma_{Y}$. We therefore allow each population in *V* to have a different variance. We need to make the distinction between *V* and *U* because the populations in *U* need not necessarily be pairwise disjoint. For example, in the main paper, subgroup and complement are in both families but the full population is only in *U*.

We now assume that in each population P∈U, we test for $r_{P}$ different candidate shapes. In each population *P* we therefore perform tests of $H_{0}^{\left(P,m\right)}:{c_{m}^{\left(P\right)}}^{{}^{\prime }}\mu^{\left(P\right)}=0$ against the alternatives $H_{1}^{\left(P,m\right)}:{c_{m}^{\left(P\right)}}^{{}^{\prime }}\mu^{\left(P\right)}>0$, for $m=1,\cdots ,r_{P}$. The test statistics for these hypotheses are then

(A.2) $$T_{m}^{\left(P\right)}=\frac{{\left(c_{m}^{\left(P\right)}\right)}^{{}^{\prime }}{\bar{Y}}^{\left(P\right)}}{\sqrt{{\left({\hat{\sigma }}^{\left(P\right)}\right)}^{2}\sum_{i=1}^{k}{c_{m}^{\left(P\right)}}^{2}/n_{i}^{\left(P\right)}}},$$

where ${\bar{Y}}^{\left(P\right)}$ is the vector of the estimated means on each dose level ${\bar{Y}}^{\left(P\right)}={\left({\bar{Y_{1}}}^{\left(P\right)},\ldots ,{\bar{Y_{k}}}^{\left(P\right)}\right)}^{{}^{\prime }}$ and ${\left({\hat{\sigma }}^{\left(P\right)}\right)}^{2}$ the estimated variances.

For the joint distribution of the test statistics, we need to derive the correlation matrix of the vector

$$T^{*}:={\left[{\left(c_{1}^{\left(P_{1}\right)}\right)}^{{}^{\prime }}{\bar{Y}}^{\left(P_{1}\right)},\cdots ,{\left(c_{r_{P_{1}}}^{\left(P_{1}\right)}\right)}^{{}^{\prime }}{\bar{Y}}^{\left(P_{1}\right)},\cdots ,{\left(c_{1}^{\left(P_{W}\right)}\right)}^{{}^{\prime }}{\bar{Y}}^{\left(P_{W}\right)},\cdots ,{\left(c_{r_{P_{W}}}^{\left(P_{W}\right)}\right)}^{{}^{\prime }}{\bar{Y}}^{\left(P_{W}\right)}\right]}^{{}^{\prime }}.$$

We denote by $\Gamma_{P}:=\left[c_{1}^{\left(P\right)},\ldots ,c_{r_{P}}^{\left(P\right)}\right]$ the matrix of optimal contrast coefficients for population *P* and by $A_{P}\in R^{kxn}$ a matrix with entries

$$\left(a_{ij}\right)=\left\{\begin{array}{cc} \frac{1}{n_{i}^{\left(P\right)}} & \text{if}\;\text{the}\;\text{jth}\;\text{patient}\;\text{is}\;\text{on}\;\text{dose}\;i\;\text{and}\;\text{is}\;\text{in}\;\text{population}\;P\\ 0 & \text{else} \end{array}\right.$$

so that $A_{P}Y={\bar{Y}}^{\left(P\right)}$. We can then write the required covariance matrix between all contrasts as

$$\begin{array}{ccc} \text{cov}(T^{*}) & = & \text{cov}\left(\left[\begin{array}{ccc} \Gamma_{P_{1}}^{{}^{\prime }} & 0 & 0\\ 0 & \ddots & 0\\ 0 & 0 & \Gamma_{P_{W}}^{{}^{\prime }} \end{array}\right]\left[\begin{array}{c} {\bar{Y}}^{\left(P_{1}\right)}\\ \vdots \\ {\bar{Y}}^{\left(P_{W}\right)} \end{array}\right]\right)=\text{cov}\left(\left[\begin{array}{ccc} \Gamma_{P_{1}}^{{}^{\prime }} & 0 & 0\\ 0 & \ddots & 0\\ 0 & 0 & \Gamma_{P_{W}}^{{}^{\prime }} \end{array}\right]\left[\begin{array}{c} A_{P_{1}}\\ \vdots \\ A_{P_{U}} \end{array}\right]Y\right)\\ & = & \left[\begin{array}{c} \Gamma_{P_{1}}^{{}^{\prime }}A_{P_{1}}\\ \vdots \\ \Gamma_{P_{W}}^{{}^{\prime }}A_{P_{W}} \end{array}\right]\Sigma_{Y}{\left[\begin{array}{c} \Gamma_{P_{1}}^{{}^{\prime }}A_{P_{1}}\\ \vdots \\ \Gamma_{P_{W}}^{{}^{\prime }}A_{P_{W}} \end{array}\right]}^{{}^{\prime }}=\left[\begin{array}{ccc} \Gamma_{P_{1}}^{{}^{\prime }}A_{P_{1}}\Sigma_{Y}A_{P_{1}}^{{}^{\prime }}\Gamma_{P_{1}}^{{}^{\prime }} & \cdots & \Gamma_{P_{1}}^{{}^{\prime }}A_{P_{1}}\Sigma_{Y}A_{P_{W}}^{{}^{\prime }}\Gamma_{P_{W}}^{{}^{\prime }}\\ \vdots & \ddots & \vdots \\ \Gamma_{P_{W}}^{{}^{\prime }}A_{P_{W}}\Sigma_{Y}A_{P_{1}}^{{}^{\prime }}\Gamma_{P_{1}}^{{}^{\prime }} & \cdots & \Gamma_{P_{W}}^{{}^{\prime }}A_{P_{W}}\Sigma_{Y}A_{P_{W}}^{{}^{\prime }}\Gamma_{P_{W}}^{{}^{\prime }} \end{array}\right]\\ & := & \left[\begin{array}{ccc} \Sigma_{P_{1}P_{1}} & \cdots & \Sigma_{P_{1}P_{W}}\\ \vdots & \ddots & \vdots \\ \Sigma_{P_{W}P_{1}} & \cdots & \Sigma_{P_{W}P_{W}} \end{array}\right]. \end{array}$$

From this covariance matrix, we can obtain the required correlation matrix ***R***:

$$R=\left[\begin{array}{ccc} R_{P_{1}P_{1}} & \cdots & R_{P_{1}P_{W}}\\ \vdots & \ddots & \vdots \\ R_{P_{W}P_{1}} & \cdots & R_{P_{W}P_{W}} \end{array}\right],$$

where the entries of the submatrix ***R_PQ_*** for the correlations between tests in populations *P* and *Q* are

$${\left(\rho_{ij}\right)}^{\left(PQ\right)}=\frac{\sum_{l=1}^{k}\left[c_{il}c_{jl}/\left(n_{l}^{\left(P\right)}n_{l}^{\left(Q\right)}\right)\sum_{P^{*}\in V}n_{l}^{\left(P\cap Q\cap P^{*}\right)}\sigma_{P^{*}}^{2}\right]}{\sqrt{\sum_{l=1}^{k}\left[{c_{il}}^{2}/{n_{l}^{\left(P\right)}}^{2}\sum_{P^{*}\in V}n_{l}^{\left(P\cap P^{*}\right)}\sigma_{P^{*}}^{2}\right]\sum_{l=1}^{k}\left[{c_{jl}}^{2}/{n_{l}^{\left(Q\right)}}^{2}\sum_{P^{*}\in V}n_{l}^{\left(Q\cap P^{*}\right)}\sigma_{P^{*}}^{2}\right]}}.$$

The cases discussed in the main part of this paper correspond to U={F,S,C} and V={S,C}.

As in the main part of the paper, the form of the joint distribution, which is used to derive critical values depends on the variance estimators in (A.2). Possible joint distributions are summarized in Table 1. In the general case, the degrees of freedom have to be adjusted for the possible higher number of populations as well.

### A.2 Simulation results for model selection

Table A.1 shows the simulation results for model selection in the case of homogeneous variances in subgroup and complement. The table shows the probability of choosing the correct model as the best model in the MCP‐step. The best model is defined as the model with the lowest *p*‐value over all tested populations.

**Table A.1:** Probability of choosing the correct dose–response model for single‐population (SP) and multipopulation (MP) testing methods

		Scenario and prevalence								
		Same	Same	Same	Double	Double	Double	Only	Only	Only
Correct model	Method	0.25	0.5	0.75	0.25	0.5	0.75	0.25	0.5	0.75
$E_{max}$	SP	0.50	0.51	0.50	0.37	0.44	0.45	0.26	0.33	0.40
	MP‐Pooled(F + S)	0.49	0.49	0.48	0.38	0.44	0.45	0.34	0.40	0.45
	MP‐Pooled(F + S + C)	0.48	0.48	0.49	0.37	0.43	0.46	0.34	0.41	0.46
Linear	SP	0.27	0.28	0.27	0.17	0.21	0.24	0.10	0.16	0.21
	MP‐Pooled(F + S)	0.27	0.27	0.27	0.17	0.21	0.24	0.18	0.20	0.24
	MP‐Pooled(F + S + C)	0.27	0.28	0.27	0.17	0.20	0.24	0.19	0.20	0.24
Exponential	SP	0.69	0.69	0.69	0.60	0.62	0.66	0.44	0.54	0.63
	MP‐Pooled(F + S)	0.68	0.69	0.68	0.58	0.62	0.67	0.54	0.60	0.65
	MP‐Pooled(F + S + C)	0.68	0.67	0.67	0.57	0.61	0.67	0.52	0.61	0.65
Logistic	SP	0.56	0.56	0.57	0.43	0.48	0.52	0.30	0.40	0.48
	MP‐Pooled(F + S)	0.56	0.55	0.57	0.42	0.48	0.52	0.38	0.46	0.52
	MP‐Pooled(F + S + C)	0.55	0.55	0.57	0.41	0.48	0.52	0.38	0.46	0.53
Quadratic	SP	0.76	0.76	0.76	0.64	0.69	0.71	0.43	0.60	0.67
	MP‐Pooled(F + S)	0.75	0.75	0.75	0.62	0.68	0.71	0.52	0.67	0.71
	MP‐Pooled(F + S + C)	0.74	0.75	0.74	0.62	0.68	0.71	0.51	0.66	0.71

*Note*. The best model is chosen based on the lowest significant *p*‐value across all populations. Probabilities shown are conditional on the global null hypothesis being rejected.
