# Supplementary material for: A multiple comparison procedure for dose‐finding trials with subpopulations
Source: Biom J. 2019 Sep 23;62(1):53–68. doi: 10.1002/bimj.201800111 (PMC6973002; doi:10.1002/bimj.201800111)
Supplement: Supplementary file 2 — Supporting Information [file BIMJ-62-53-s001.pdf]

Supporting Information for the article 'A multiple comparison procedure for dose-finding trials with subpopulations'

Contents

1 Additional simulation results for homoscedastic scenarios **2**

1.1 Results for other data-generating models 2

1.2 Detailed power curves 5

2 Additional simulation results for heteroscedastic scenarios **8**

2.1 Comparison of approximate tests for other data-generating models 8

2.2 Comparison of multi-population MCP to single population MCP for other data-generating models 12

1 Additional simulation results for homoscedastic scenarios

1.1 Results for other data-generating models

Method	global	F	S	C
SP	0.05	0.05	-	-
MP-Pooled(F+S)	0.05	0.03	0.03	-
MP-Pooled(F+S+C)	0.05	0.03	0.02	0.02
MP-MultDF(F+S)	0.05	0.03	0.02	-
MP-MultDF(F+S+C)	0.05	0.02	0.02	0.02

Table 1: Probability to reject the global null hypothesis and population null hypotheses for single population (SP) and multi-population (MP) testing methods. Data are generated from a constant model under homoscedasticity.

		Scenario and Prevalence								
		same	*same*	*same*	*double*	*double*	*double*	*only*	*only*	*only*
Method	Population	0.25	0.5	0.75	0.25	0.5	0.75	0.25	0.5	0.75
SP	global	0.88	0.88	0.87	0.54	0.67	0.79	0.16	0.40	0.66
MP-Pooled(F+S)	global	0.82	0.84	0.85	0.54	0.69	0.80	0.33	0.59	0.75
	F	0.81	0.82	0.83	0.43	0.58	0.73	0.10	0.32	0.60
	S	0.30	0.55	0.72	0.31	0.54	0.73	0.29	0.54	0.72
MP-Pooled(F+S+C)	global	0.82	0.82	0.82	0.51	0.64	0.75	0.30	0.52	0.69
	F	0.78	0.78	0.78	0.39	0.52	0.67	0.09	0.26	0.52
	S	0.27	0.49	0.65	0.28	0.48	0.66	0.26	0.48	0.65
	C	0.65	0.46	0.26	0.20	0.14	0.09	0.02	0.02	0.02
MP-MultDF(F+S)	global	0.83	0.85	0.87	0.53	0.69	0.81	0.35	0.58	0.75
	F	0.81	0.83	0.85	0.43	0.59	0.74	0.11	0.31	0.59
	S	0.28	0.55	0.74	0.29	0.55	0.72	0.31	0.54	0.72
MP-MultDF(F+S+C)	global	0.83	0.83	0.83	0.50	0.65	0.75	0.31	0.52	0.68
	F	0.78	0.79	0.79	0.39	0.53	0.67	0.09	0.26	0.51
	S	0.25	0.49	0.66	0.26	0.49	0.65	0.27	0.48	0.65
	C	0.66	0.48	0.26	0.20	0.13	0.09	0.02	0.02	0.02

Table 2: Probability to reject the global null hypothesis and population null hypotheses for single population (SP) and multi-population (MP) testing methods. Data are generated from a linear model under homoscedasticity.

Method	Population	Scenario and Prevalence *same* 0.25	*same* 0.5	*same* 0.75	*double* 0.25	*double* 0.5	*double* 0.75	*only* 0.25	*only* 0.5	*only* 0.75
SP	global	0.86	0.86	0.86	0.51	0.65	0.76	0.16	0.36	0.64
MP-Pooled(F+S)	global	0.80	0.82	0.84	0.50	0.66	0.78	0.31	0.55	0.73
	F	0.78	0.80	0.82	0.41	0.56	0.70	0.10	0.29	0.58
	S	0.26	0.52	0.70	0.28	0.51	0.70	0.27	0.51	0.69
MP-Pooled(F+S+C)	global	0.80	0.80	0.80	0.47	0.61	0.72	0.28	0.49	0.67
	F	0.75	0.75	0.76	0.37	0.50	0.63	0.08	0.24	0.49
	S	0.23	0.45	0.63	0.24	0.44	0.63	0.24	0.45	0.63
	C	0.62	0.43	0.23	0.18	0.14	0.09	0.02	0.02	0.02
MP-MultDF(F+S)	global	0.81	0.82	0.85	0.49	0.66	0.79	0.30	0.55	0.74
	F	0.80	0.80	0.83	0.40	0.56	0.71	0.10	0.28	0.58
	S	0.27	0.52	0.71	0.26	0.50	0.71	0.26	0.51	0.71
MP-MultDF(F+S+C)	global	0.81	0.81	0.81	0.46	0.61	0.72	0.26	0.49	0.67
	F	0.76	0.76	0.77	0.36	0.50	0.64	0.08	0.23	0.49
	S	0.24	0.46	0.64	0.23	0.44	0.63	0.22	0.45	0.63
	C	0.62	0.44	0.24	0.18	0.13	0.09	0.02	0.02	0.02

Table 3: Probability to reject the global null hypothesis and population null hypotheses for single population (SP) and multi-population (MP) testing methods. Data are generated from an exponential model under homoscedasticity.

Method	Population	Scenario and Prevalence *same* 0.25	*same* 0.5	*same* 0.75	*double* 0.25	*double* 0.5	*double* 0.75	*only* 0.25	*only* 0.5	*only* 0.75
SP	global	0.95	0.95	0.95	0.64	0.80	0.88	0.20	0.49	0.78
MP-Pooled(F+S)	global	0.92	0.93	0.94	0.64	0.81	0.90	0.42	0.70	0.87
	F	0.92	0.92	0.93	0.54	0.72	0.84	0.13	0.41	0.73
	S	0.38	0.66	0.85	0.39	0.67	0.85	0.38	0.67	0.85
MP-Pooled(F+S+C)	global	0.92	0.92	0.92	0.61	0.77	0.86	0.38	0.65	0.82
	F	0.90	0.90	0.90	0.50	0.67	0.80	0.11	0.36	0.66
	S	0.34	0.60	0.80	0.35	0.61	0.80	0.34	0.61	0.79
	C	0.80	0.60	0.35	0.27	0.19	0.11	0.02	0.02	0.02
MP-MultDF(F+S)	global	0.92	0.93	0.94	0.63	0.81	0.90	0.41	0.72	0.86
	F	0.92	0.92	0.93	0.53	0.72	0.85	0.13	0.41	0.73
	S	0.39	0.68	0.84	0.37	0.67	0.84	0.37	0.68	0.84
MP-MultDF(F+S+C)	global	0.92	0.92	0.92	0.60	0.76	0.86	0.37	0.66	0.81
	F	0.90	0.90	0.90	0.49	0.66	0.80	0.11	0.35	0.66
	S	0.34	0.62	0.78	0.32	0.61	0.79	0.33	0.62	0.79
	C	0.80	0.60	0.34	0.25	0.20	0.11	0.02	0.02	0.02

Table 4: Probability to reject the global null hypothesis and population null hypotheses for single population (SP) and multi-population (MP) testing methods. Data are generated from a logistic model under homoscedasticity.

Method	Population	Scenario and Prevalence								
		same	*same*	*same*	*double*	*double*	*double*	*only*	*only*	*only*
		0.25	0.5	0.75	0.25	0.5	0.75	0.25	0.5	0.75
SP	global	0.79	0.80	0.79	0.43	0.57	0.68	0.14	0.31	0.54
MP-Pooled(F+S)	global	0.73	0.75	0.77	0.42	0.58	0.70	0.26	0.47	0.65
	F	0.71	0.72	0.74	0.33	0.48	0.62	0.08	0.24	0.48
	S	0.22	0.42	0.61	0.23	0.43	0.62	0.23	0.43	0.61
MP-Pooled(F+S+C)	global	0.72	0.71	0.72	0.39	0.53	0.64	0.24	0.41	0.57
	F	0.67	0.66	0.67	0.29	0.42	0.54	0.07	0.20	0.40
	S	0.19	0.37	0.53	0.20	0.37	0.54	0.20	0.37	0.54
	C	0.53	0.36	0.21	0.15	0.11	0.07	0.02	0.02	0.02
MP-MultDF(F+S)	global	0.72	0.74	0.78	0.42	0.58	0.69	0.25	0.47	0.65
	F	0.70	0.72	0.74	0.33	0.48	0.61	0.08	0.24	0.49
	S	0.23	0.42	0.62	0.23	0.44	0.60	0.22	0.43	0.61
MP-MultDF(F+S+C)	global	0.72	0.72	0.73	0.40	0.53	0.62	0.23	0.41	0.58
	F	0.66	0.67	0.67	0.29	0.42	0.53	0.07	0.20	0.40
	S	0.20	0.36	0.54	0.19	0.38	0.52	0.19	0.37	0.54
	C	0.52	0.36	0.20	0.14	0.11	0.07	0.02	0.02	0.03

Table 5: Probability to reject the global null hypothesis and population null hypotheses for single population (SP) and multi-population (MP) testing methods. Data are generated from a quadratic model under homoscedasticity.

1.2 Detailed power curves

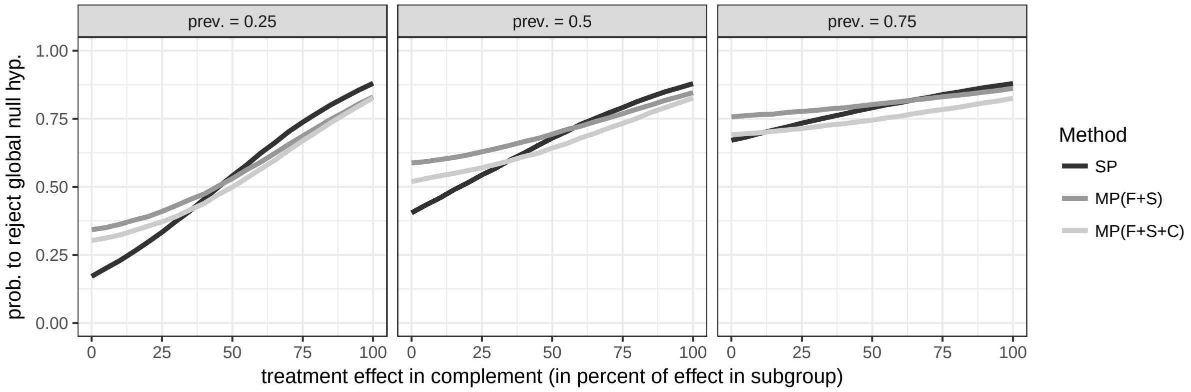

Figure 1: Probability to reject the global null hypothesis for single population (SP) and multi-population (MP) testing methods plotted against the treatment effect in the complement in relation to the subgroup. Data are generated from an Emax model under homoscedasticity.

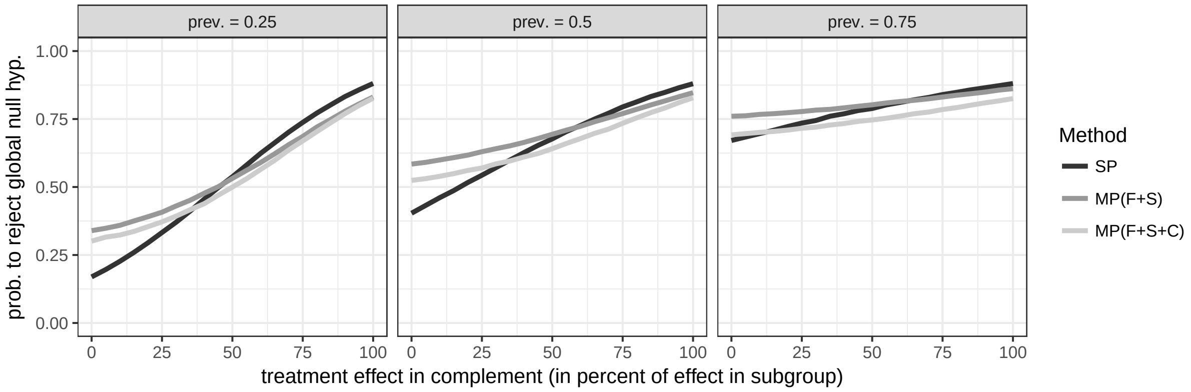

Figure 2: Probability to reject the global null hypothesis for single population (SP) and multi-population (MP) testing methods plotted against the treatment effect in the complement in relation to the subgroup. Data are generated from a linear model under homoscedasticity.

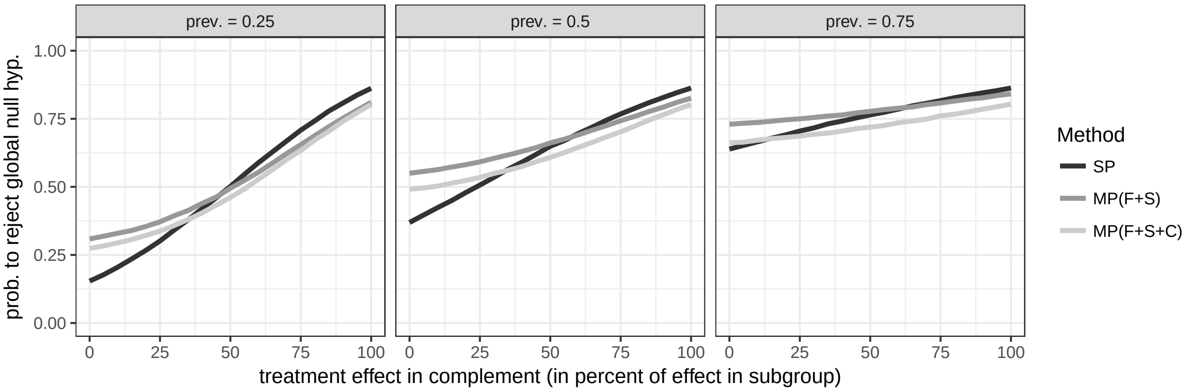

Figure 3: Probability to reject the global null hypothesis for single population (SP) and multi-population (MP) testing methods plotted against the treatment effect in the complement in relation to the subgroup. Data are generated from an exponential model under homoscedasticity.

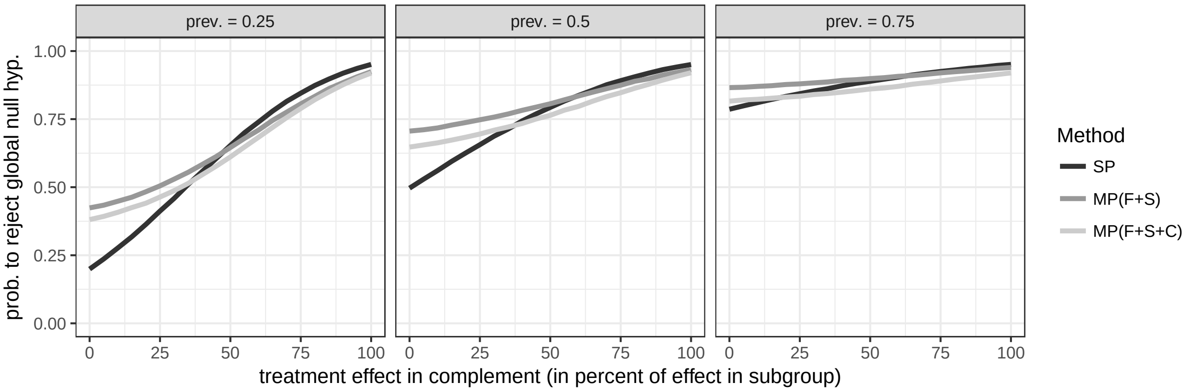

Figure 4: Probability to reject the global null hypothesis for single population (SP) and multi-population (MP) testing methods plotted against the treatment effect in the complement in relation to the subgroup. Data are generated from a logistic model under homoscedasticity.

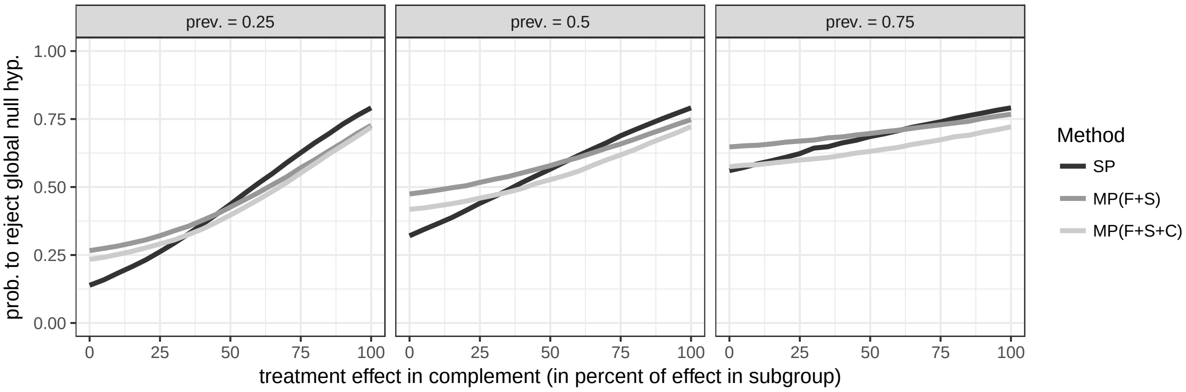

Figure 5: Probability to reject the global null hypothesis for single population (SP) and multi-population (MP) testing methods plotted against the treatment effect in the complement in relation to the subgroup. Data are generated from a quadratic model under homoscedasticity.

2 Additional simulation results for heteroscedastic scenarios

2.1 Comparison of approximate tests for other data-generating models

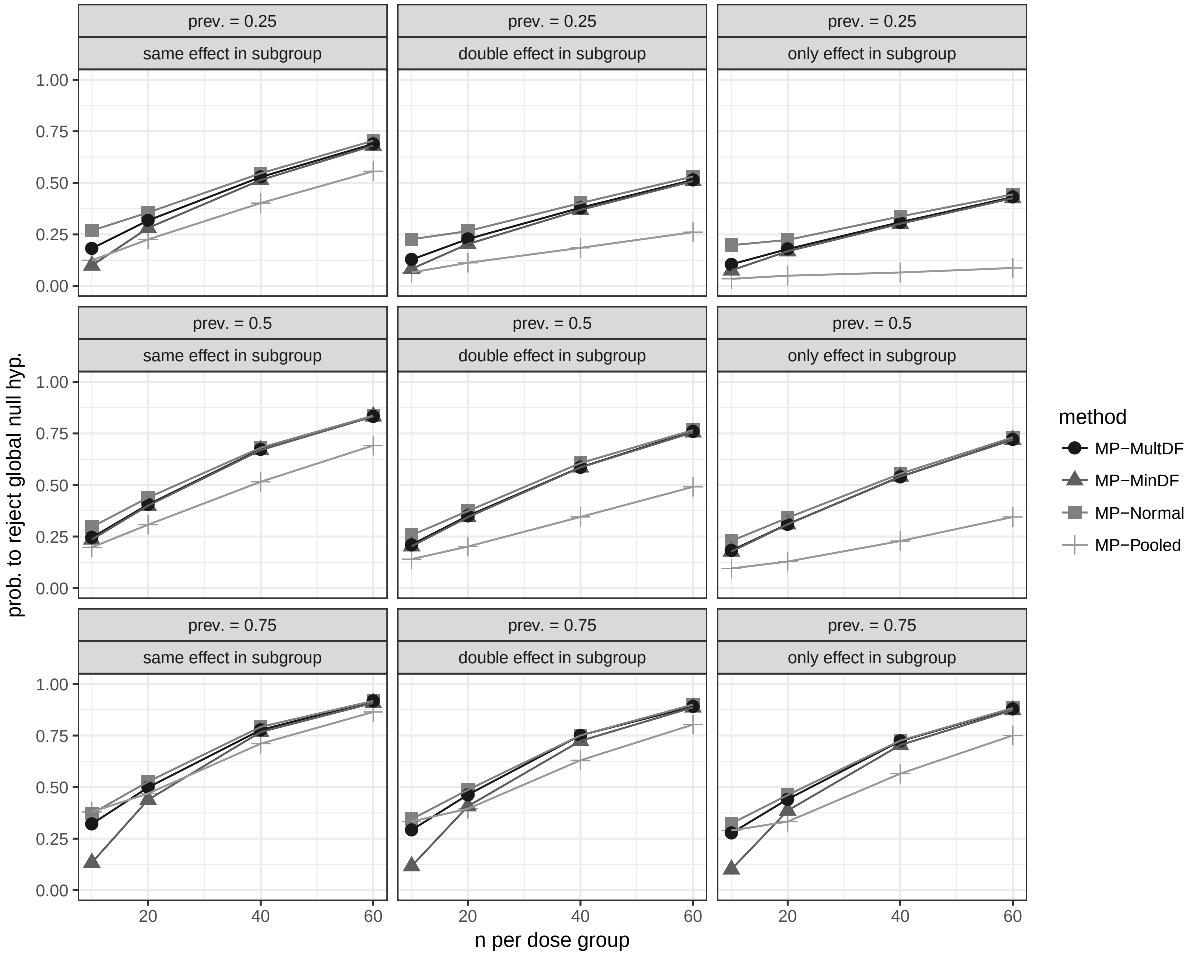

Figure 6: Probability to reject the global null hypothesis for single population (SP) and multi-population (MP) testing methods. Data are generated from a linear model under heteroscedasticity. MP-MultDF is used to approximate the joint distribution for MP testing methods.

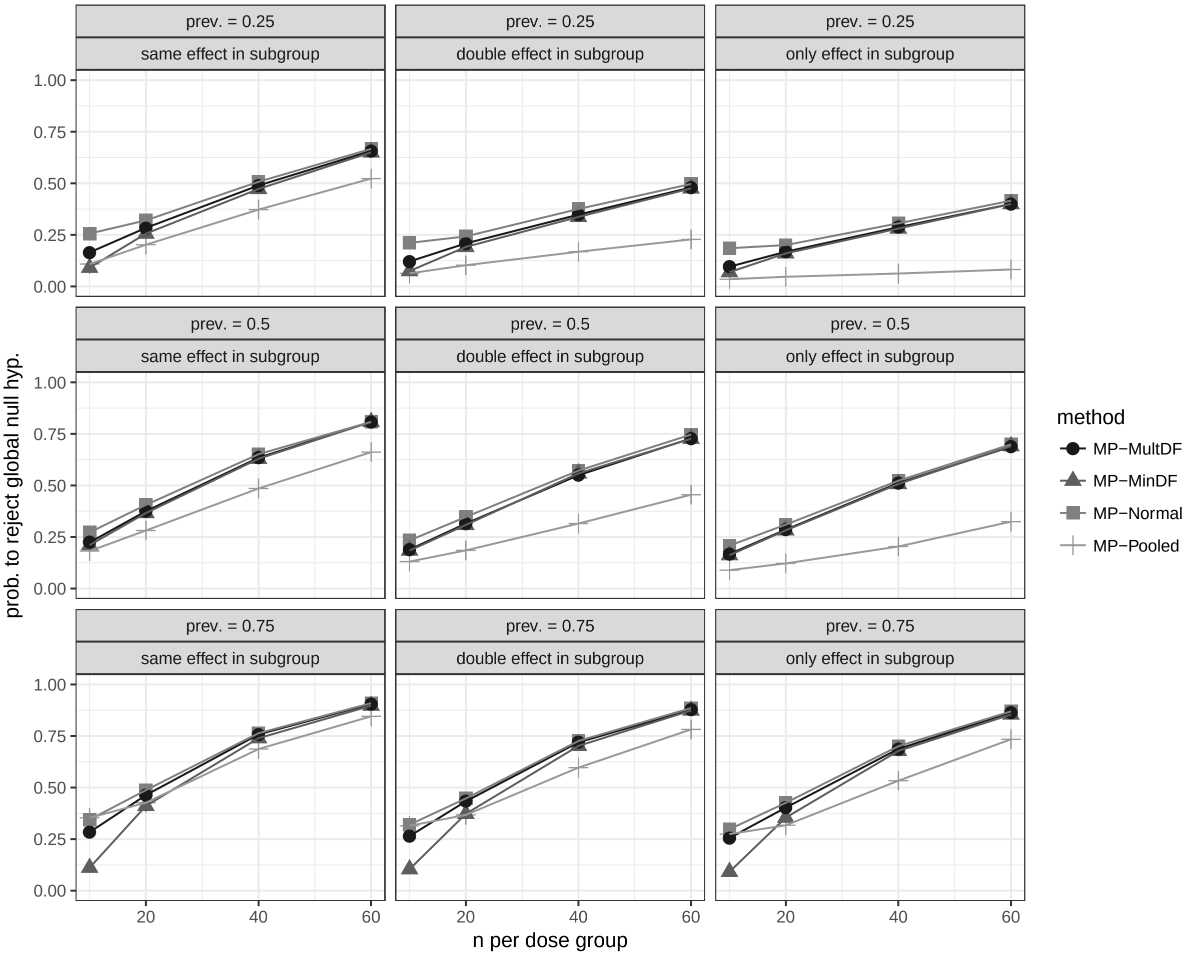

Figure 7: Probability to reject the global null hypothesis for single population (SP) and multi-population (MP) testing methods. Data are generated from an exponential model under heteroscedasticity. MP-MultDF is used to approximate the joint distribution for MP testing methods.

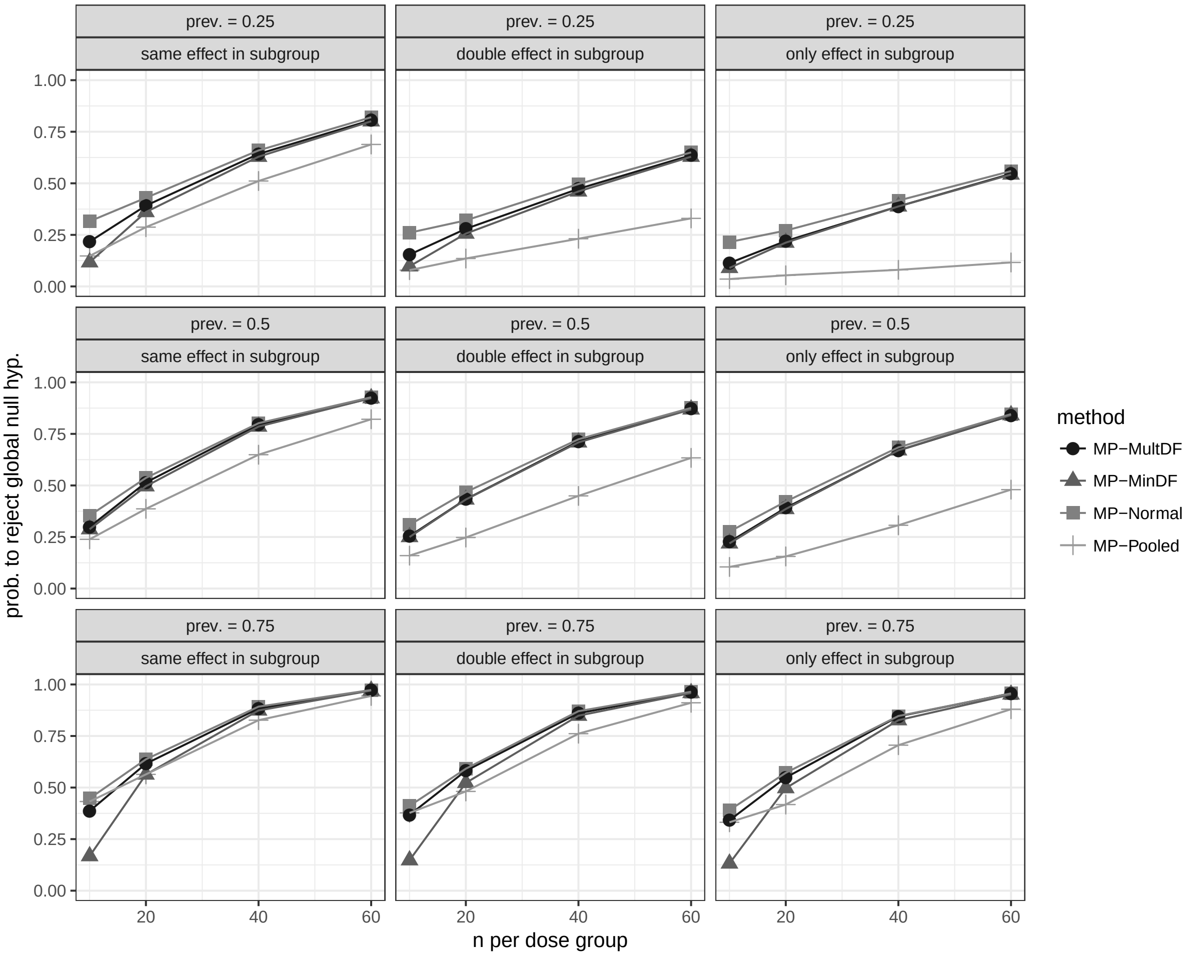

Figure 8: Probability to reject the global null hypothesis for single population (SP) and multi-population (MP) testing methods. Data are generated from a logistic model under heteroscedasticity. MP-MultDF is used to approximate the joint distribution for MP testing methods.

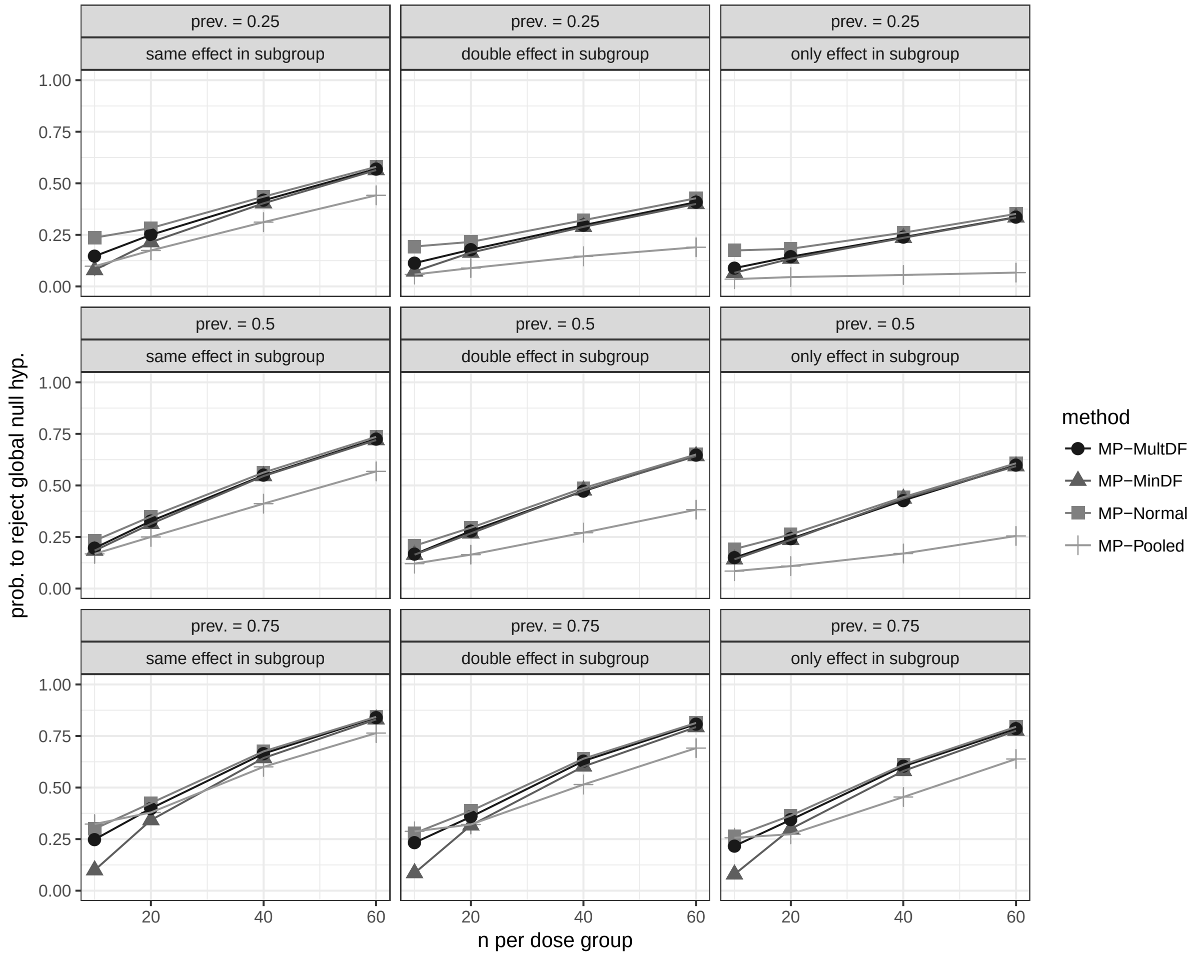

Figure 9: Probability to reject the global null hypothesis for single population (SP) and multi-population (MP) testing methods. Data are generated from a quadratic model under heteroscedasticity. MP-MultDF is used to approximate the joint distribution for MP testing methods.

2.2 Comparison of multi-population MCP to single population MCP for other data-generating models

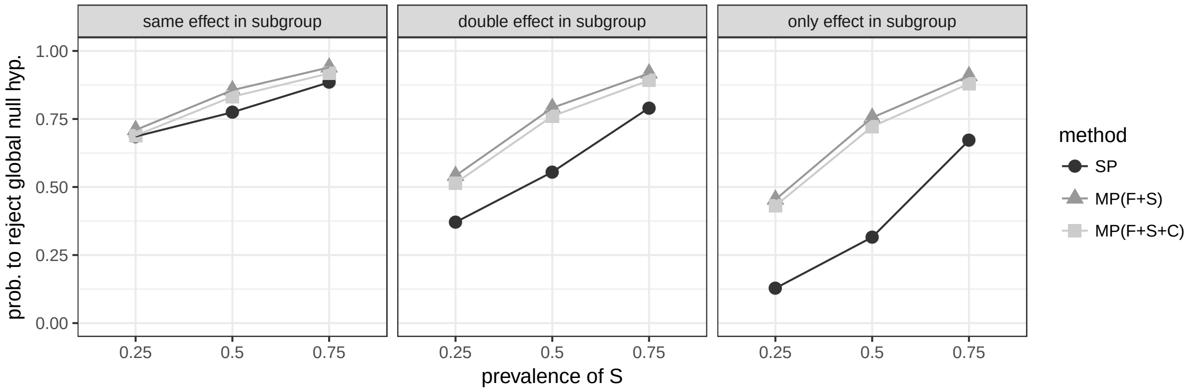

Figure 10: Probability to reject the global null hypothesis for single population (SP) and multi-population (MP) testing methods. Data are generated from a linear model under heteroscedasticity. MP-MultDF is used to approximate the joint distribution for MP testing methods.

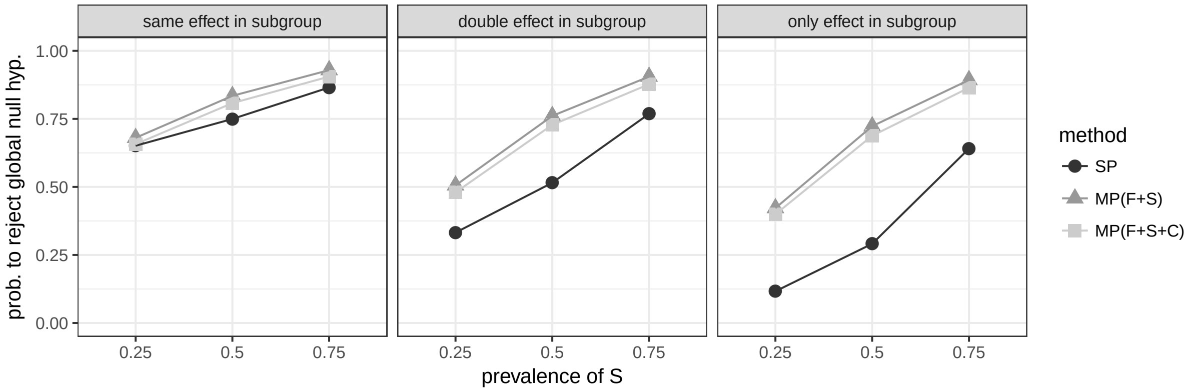

Figure 11: Probability to reject the global null hypothesis for single population (SP) and multi-population (MP) testing methods. Data are generated from an exponential model under heteroscedasticity. MP-MultDF is used to approximate the joint distribution for MP testing methods.

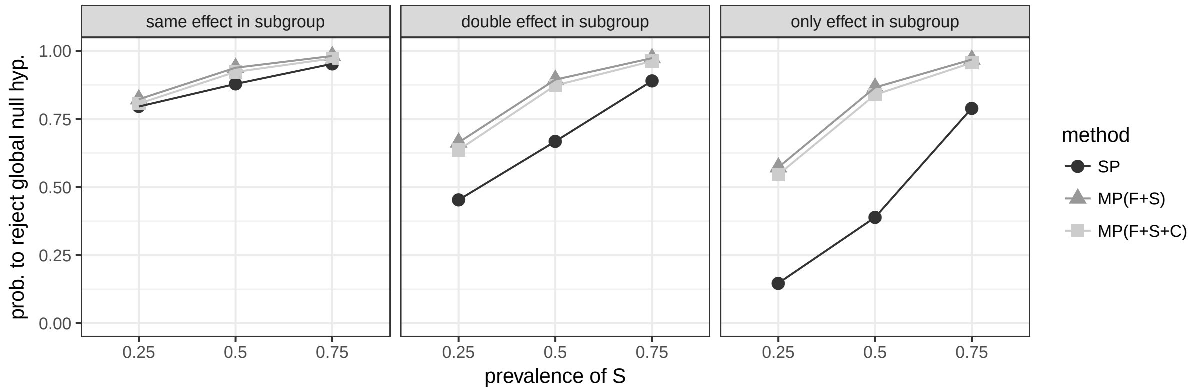

Figure 12: Probability to reject the global null hypothesis for single population (SP) and multi-population (MP) testing methods. Data are generated from a logistic model under heteroscedasticity. MP-MultDF is used to approximate the joint distribution for MP testing methods.

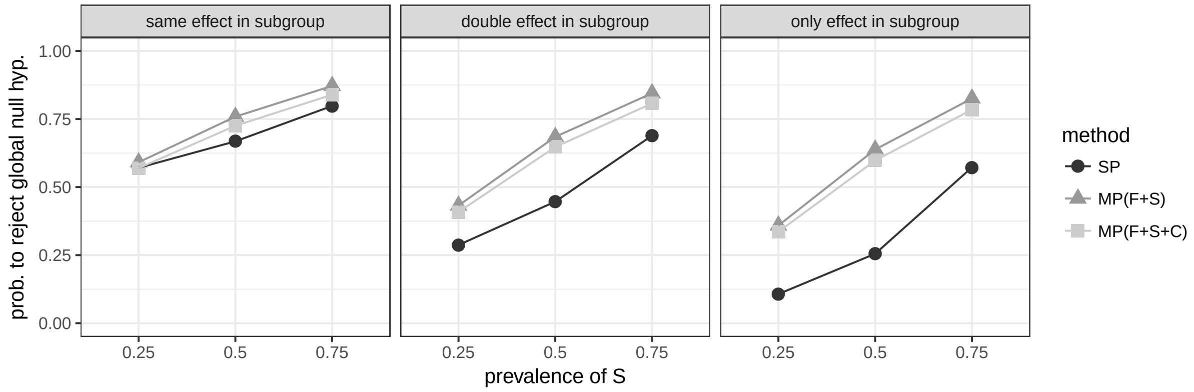

Figure 13: Probability to reject the global null hypothesis for single population (SP) and multi-population (MP) testing methods. Data are generated from a quadratic model under heteroscedasticity. MP-MultDF is used to approximate the joint distribution for MP testing methods.
